# A Comparison of Photoprotective Mechanism in Different Light-Demanding Plants Under Dynamic Light Conditions

**DOI:** 10.3389/fpls.2022.819843

**Published:** 2022-04-06

**Authors:** Sheng-Pu Shuang, Jin-Yan Zhang, Zhu Cun, Hong-Min Wu, Jie Hong, Jun-Wen Chen

**Affiliations:** ^1^College of Agronomy and Biotechnology, Yunnan Agricultural University, Kunming, China; ^2^Key Laboratory of Medicinal Plant Biology of Yunnan Province, Yunnan Agricultural University, Kunming, China; ^3^National and Local Joint Engineering Research Center on Germplasm Innovation and Utilization of Chinese Medicinal Materials in Southwestern China, Yunnan Agricultural University, Kunming, China

**Keywords:** cyclic electron flow, dynamic light, light energy distribution, non-photochemical quenching, photoprotection

## Abstract

Light intensity is highly heterogeneous in nature, and plants have evolved a series of strategies to acclimate to dynamic light due to their immobile lifestyles. However, it is still unknown whether there are differences in photoprotective mechanisms among different light-demanding plants in response to dynamic light, and thus the role of non-photochemical quenching (NPQ), electron transport, and light energy allocation of photosystems in photoprotection needs to be further understood in different light-demanding plants. The activities of photosystem II (PSII) and photosystem I (PSI) in shade-tolerant species *Panax notoginseng*, intermediate species *Polygonatum kingianum*, and sun-demanding species *Erigeron breviscapus* were comparatively measured to elucidate photoprotection mechanisms in different light-demanding plants under dynamic light. The results showed that the NPQ and PSII maximum efficiency (*F*_v_′/*F*_m_′) of *E. breviscapus* were higher than the other two species under dynamic high light. Meanwhile, cyclic electron flow (CEF) of sun plants is larger under transient high light conditions since the slope of post-illumination, P700 dark reduction rate, and plastoquinone (PQ) pool were greater. NPQ was more active and CEF was initiated more readily in shade plants than the two other species under transient light. Moreover, sun plants processed higher quantum yield of PSII photochemistry (Φ_PSII_), quantum yield of photochemical energy conversion [Y(I)], and quantum yield of non-photochemical energy dissipation due to acceptor side limitation (Y(NA), while the constitutive thermal dissipation and fluorescence (Φ_f,d_) and quantum yield of non-photochemical energy dissipation due to donor side limitation [Y(ND)] of PSI were higher in shade plants. These results suggest that sun plants had higher NPQ and CEF for photoprotection under transient high light and mainly allocated light energy through Φ_PSII_ and Φ_NPQ_, while shade plants had a higher Φ_f,d_ and a larger heat dissipation efficiency of PSI donor. Overall, it has been demonstrated that the photochemical efficiency and photoprotective capacity are greater in sun plants under transient dynamic light, while shade plants are more sensitive to transient dynamic light.

## Introduction

Light is the source of energy for photosynthesis and influences the growth and development of plants (Chen et al., 2016). However, light intensity is highly dynamic on both temporal and spatial scales (Rascher and Nedbal, 2006; Hallik et al., 2011; Liu and Last, 2017). Therefore, plants have to acclimate to high light heterogeneity due to their immobile lifestyles (Chen et al., 2016), and thus plants have long evolved into shade species, intermediate species, and sun species (Augspurger, 1984; Kitajima, 1994; Poorter et al., 2019). Sun plants *Tectona grandis* and *Phyllostachys heterocycla* have higher maximum net photosynthetic rates (*P*_n–max_) and light saturation points (LSP) than the shade plant *Commelina purpurea* (Valladares et al., 2000; Valladares and Niinemets, 2008; Mishanin et al., 2017). Shade plants readily suffer photoinhibition and even photodamage because of their low photosynthetic capacity and LSP under natural conditions (Boardman, 1977; Lichtenthaler and Babani, 2004; Way and Pearcy, 2012; Huang et al., 2015); correspondingly, sun plants confront a lesser photooxidative pressure.

The excess light energy might cause the photosystem to be restrained, reduce carbon assimilation, and even cause plant death in severe cases (Niyogi and Truong, 2013; Vialet-Chabrand et al., 2017; Wei et al., 2020). Non-photochemical quenching (NPQ) of photosystem II (PSII) is considered to be the most effective first line of defense in dissipating excess light energy (Demmig-Adams et al., 2012; Wei et al., 2020). PSII of shade plant *Amomum villosum* and sun plants *Anthocephalus chinensis* and *Barringtonia racemosa* is protected by a high NPQ to escape from photoinhibition in high light (Feng et al., 2001, 2002). PsbS and the xanthophyll cycle play an important role in regulating the NPQ process as energy quenching catalysts (Hubbart et al., 2012; Suorsa et al., 2012; Ikeuchi et al., 2014), and the PsbS largely determines the rate of NPQ induction/relaxation (Li et al., 2000, 2004; Horton et al., 2008; Johnson and Ruban, 2010, 2011). Violaxanthin (V) is de-epoxied to form antheraxanthin (A) when *Arabidopsis thaliana* is exposed to high light, which is further de-epoxied to form zeaxanthin (Z) (Garcia-Molina and Leister, 2020). Z is used as the quenching site of excess excitation energy to dissipate excess light energy (Jahns and Holzwarth, 2012). NPQ is positively correlated with the xanthophylls de-epoxidation state (DES) under steady-state light; however, it is still unknown whether the V cycle is activated in different light-demanding plants and it relates to NPQ. Photosystem I (PSI) of sun plant *A. thaliana* is suppressed under dynamic light conditions, resulting in decreased growth rate and biomass accumulation (Suorsa et al., 2012; Vialet-Chabrand et al., 2017; Schneider et al., 2019). Dynamic light-induced photoinhibition of PSI has also been recorded in sun plants *Helianthus annuus*, *Triticum aestivum*, and *Oryza sativa* (Takehiro et al., 2014; Zivcak et al., 2015; Yamori et al., 2016). Light energy absorbed by plants is allocated into the quantum yield of ΔpH and xanthophyll-regulated thermal energy dissipation (Φ_NPQ_), the quantum yield of PSII photochemistry (Φ_PSII_), and constitutive thermal dissipation and fluorescence (Φ_f,d_). Three pathways for the allocation of light energy are competitive, but there is a trade-off among them (Hendrickson et al., 2004; Kornyeyev and Hendrickson, 2007; Farooq et al., 2018). A larger proportion of Φ_PSII_ and Φ_NPQ_ has been observed in sun plants *Vitis vinifera* and *O. sativa* under stable and continuous high light conditions (Hendrickson et al., 2004; Ishida et al., 2014). Sun plant *Magnolia grandiflora* has a high photochemical and thermal dissipation capacity by reducing the chlorophyll content and the number of photopigment molecules under short-term intense light irradiation (Valladares et al., 2002). Shade plant *Psychotria asiatica* exposed to full light exhibits a high P700 oxidation rate to regulate energy distribution (Huang et al., 2015). Therefore, the light energy distribution is important for different light-demanding plants to mitigate photodamage caused by unfavorable light. At present, relatively less is known about the allocation and balance of light energy absorbed by PSII and PSI in different light-demanding plants in response to different intensities of transient light.

Plants would inevitably suffer oversaturation of photosystem electron transport if the excess light energy is dissipated only through the NPQ pathway. Cyclic electron flow (CEF) is an efficient alternative pathway for expending photosynthetic electrons (Tikhonov, 2013; Ishida et al., 2014). In angiosperms, PSI cycle electron transport can be mediated through two known pathways (Yamori et al., 2016). One is to depend on proton gradient regulation 5 (PGR5) and PGR like 1 (PGRL1) (Munekage et al., 2002; DalCorso et al., 2008). The other is mediated by chloroplast NAD(P)H dehydrogenase (NDH) (Burrows et al., 1998). The CEF-dependent generation of the proton gradient (ΔpH) across the thylakoid membrane not only stimulates ATP synthesis but also protects photosystem II from photoinhibition through activating NPQ and stabilizing oxygen-evolving complexes (Huang et al., 2012a,^2015^). Furthermore, CEF can also alleviate the over-reduction of PSI electron acceptors, reduce the synthesis of superoxide anions in PSI, and prevent PSI from photoinhibition and photooxidative damage (Huang et al., 2012a). Once sun plants *Nicotiana tabacum* and *O. sativa* are exposed to constant high light conditions, CEF regulates electron transport *via* Cytb6/f complex and protects PSII and PSI from photooxidative damage (Miyake, 2010; Kubo et al., 2011; Satoshi et al., 2014). The NDH-mediated CEF plays a protective role in the exposure to the shade plant *Marchantia polymorpha* to high light (Ueda et al., 2012). The photoinhibition mitigation of PSI donor and acceptor side could be achieved by CEF initiation in sun plant *Cerasus cerasoides* and intermediate plant *Bletilla striata* grown under fluctuating light (Yang et al., 2019a,b). However, a comparative investigation on the photoprotection of PSI is relatively fewer across the different light-demanding plants under dynamic light conditions.

*Panax notoginseng* is a typical shade plant of the *Panax* genus in the Araliaceae family, which is commonly planted in a shaded environment constructed by shade nets in production (Xu et al., 2018; Zhang et al., 2020). It has a vigorous photosynthetic capacity and a high accumulation of secondary metabolites under light transmittance of 9.6–11.5% (Chen et al., 2014, 2016; Xu et al., 2018; Zhang et al., 2020). *Polygonatum kingianum* is a perennial plant belonging to the *Polygonatum* genus of the Liliaceae family. Light requirements are intermediate for *P. kingianum* that might grow at open or closed sites (Yao et al., 2018; Zuo et al., 2018). The light compensation point of *P. kingianum* is lower than 20 μmol m^–2^ s^–1^, and its light saturation point is higher than 1,000 μmol m^–2^ s^–1^, indicating that *P. kingianum* not only tolerates low light but also adapts to high light (Yao et al., 2018). *Erigeron breviscapus* is a perennial herb of the *Erigeron* genus in the Asteraceae family and is a native medicinal herb of Yunnan, China (Li et al., 2019). The biomass accumulation of *E. breviscapus* is highest under full sunlight conditions, while shade considerably decreases its biomass (Su et al., 2006). In this study, *P. notoginseng*, *P. kingianum*, and *E. breviscapus* were selected to explore the photoprotection of three different light-demanding plants under dynamic light of different intensities. We anticipated the following: (1) The NPQ of shade plants is more active under transient low light, and the NPQ of sun plants is more efficient under transient high light; (2) Under dynamic light, the sun plant allocates a higher proportion of light energy to the photosynthetic pathway, and the shade plant has a higher proportion of Φ_f,d_; and (3) the CEF of shade plants is initiated more readily than the CEF of sun plants under dynamic light of different intensities.

## Materials and Methods

### Plant Materials and Growth Conditions

The experimental site was located in the teaching and experimental farm of Yunnan Agricultural University in Kunming, Yunnan Province, China (altitude: 1,976 m; 102°45′32′′ E; 25°8′2′′ N; annual rainfall: 1,000 mm; and annual average temperature: 15.1°C). The rainy season is from May to October, and the dry season is from November to April in the following year. In this study, *P. notoginseng* (Burk.) F. H. Chen, *P. kingianum* Coll. Et Hemsl, and *E. breviscapus* (Vant.) Hand. -Mazz. were selected as experimental materials. *P. notoginseng* is a typical shade-tolerant species, and it commonly grows under about 10% of full sunlight. *P. kingianum* is an intermediate species, and *E. breviscapus* is a sun-demanding species that grows at an open site. Plants of *P. notoginseng* were planted in a shade condition (10% of full sunlight). Plants of *P. kingianum* were planted in a moderate shading condition (70% of full sunlight). Plants of *E. breviscapus* were planted in full sun condition. A total of five independent plots were designed for each species, and at least one plant from each plot was used to measure photosynthetic-related parameters. In August 2020, the healthy mature leaves of *P. notoginseng* were selected for experiments. For *P. kingianum*, the healthy mature leaves were used for measurements. In addition, *E. breviscapus* was used for experiments with the fully expanded rosette leaves. All measurements were made using the *in situ* method.

### P700 and Chlorophyll Fluorescence Measurement

According to the method described by Kramer et al. (2004), PSI and PSII parameters were simultaneously recorded using Dual-PAM-100 (Heinz Walz, Effeltrich, Germany). After dark adaptation for 1 h, the minimal fluorescence from dark-adapted leaf (*F*_0_) and maximal fluorescence from dark-adapted leaf (*F*_m_) were measured at 0 μmol m^–2^ s^–1^ light intensity. Then three different light-demanding plants were suddenly exposed to simulated sunflecks for 30 min with a light intensity of 50, 100, 400, 800, and 1,600 μmol m^–2^ s^–1^, respectively, followed by dark treatment for 15 min, and the whole process lasted for 45 min.

The minimal fluorescence from light-adapted leaf (*F*_0_′), maximal fluorescence from light-adapted leaf (*F*_m_′), and chlorophyll stable fluorescence (*F*_s_) after light adaptation was recorded at intervals of 30 s in the first 1 min, then at intervals of 1 min. *F*_m_ and *F*_m_′ were determined by applying a saturation pulse (300 ms and 20,000 μmol m^–2^ s^–1^). Under each light intensity treatment, 5 healthy plants were selected to measure. The fluorescence parameters of PSII are calculated as follows (Genty et al., 1989; van Kooten and Snel, 1990; Demmig-Adams et al., 1996; Hendrickson et al., 2004): PSII maximum efficiency: *F*_v_′/*F*_m_′ = (*F*_m_′-*F*_0_′)/*F*_m_′; non-photochemical quenching: NPQ = (*F*_m_-*F*_m_′)/*F*_m_′; photochemical quenching: qP = (*F*_m_′-*F*_s_)/(*F*_m_′-*F*_0_′); quantum yield of PSII photochemistry: Φ_PSII_ = Y(II) = (*F*_m_′-*F*_s_)/*F*_m_′; constitutive thermal dissipation and fluorescence: Φ_f,d_ = Y(NO) = *F*_s_/*F*_m_; quantum yield of ΔpH and xanthophyll-regulated thermal energy dissipation: Φ_NPQ_ = Y(NPQ) = *F*_s_/*F*_m_′-*F*_s_/*F*_m_; the electron transport rate in PSII: ETR(II) = Y(II) × PPFD × 0.84 × 0.5, where 0.84 is the light absorption coefficient of the leaf (Ehleringer and Pearcy, 1983) and 0.5 is the ratio of light energy distributed in PSI and PSII (Major and Dunton, 2002). Calculation of *F*_v_′/*F*_m_′ induction-related traits (T_30_, T_50_, T_90_, IS_300S_, IS_600S_, and IS_1200S_) in different light-demanding plants are shown in Supplementary Figure 1.

The PSI photosynthetic parameters were measured according to the method described by Huang et al. (2012b). The P700^+^ signals (*P*) could vary between a minimum (P700 fully reduced) and a maximum level (P700 fully oxidized). The PSI reaction center P700 maximum fluorescence signal (*P*_m_) was determined by applying a saturation pulse (300 ms and 20,000 μmol m^–2^ s^–1^) after pre-illumination with far-red (FR) light for 10 s. The PSI reaction center P700 maximum fluorescence (*P*_m_′) was obtained similarly, except that actinic light was used instead of FR light. Calculation of PSI parameters included the following (Miyake et al., 2005): quantum yield of photochemical energy conversion: Y(I) = (*P*_m_′-*P*)/*P*_m_; quantum yield of non-photochemical energy dissipation due to donor side limitation: Y(ND) = 1-P700red = *P*/*P*_m_; quantum yield of non-photochemical energy dissipation due to acceptor side limitation: Y(NA) = (*P*_m_-*P*_m_′)/*P*_m_; the electron transport rate in PSI: ETR(I) = Y(I) × PPFD × 0.84 × 0.5. The value of cyclic electron transport (CEF) was estimated as ETR(I)-ETR(II) (Huang et al., 2012b,^2015^; Zivcak et al., 2013). Notably, the distribution of absorbed light energy between PSI and PSII may differ under various environmental conditions, which can lead to the possible impreciseness of ETR(I), ETR(II), and CEF.

### Measurement of Post-illumination Fluorescence

According to the method described by Wang et al. (2017), post-illumination chlorophyll fluorescence (CEF around PSI) was measured using the automatic program of the Dual-PAM software. After dark adaptation for 1 h, post-illumination fluorescence was monitored by the transient increase of dark-level chlorophyll fluorescence after actinic light (AL) illumination (50, 100, 400, 800, and 1,600 μmol m^–2^ s^–1^, respectively) had been turned off using a Dual-PAM-100 (Heinz Walz, Effeltrich, Germany).

### Kinetic Measurement of the Dark Re-reduction of P700^+^ and Photochemical Sequencing Pools

The redox state of P700 was measured using the automatic program of the Dual-PAM software according to the method described by Klughammer and Schreiber (2008). In the presence of FR light, the PQ pool was measured during a single turnover (ST) flash (PQ pools being oxidized) followed by multiple turnover (MT) flashes (PQ pools being fully reduced). The complementary area between the oxidation curve of P700 excited by ST and MT and the stable level of P700^+^ under FR represents the ST and MT areas, respectively (Savitch et al., 2001). The ratio of MT area/ST area was used to estimate the size of the PQ pool (Savitch et al., 2001).

The kinetic curve of the dark re-reduction of P700^+^ is the P700 signal changes after the FR light is turned on and off, which reflects the dynamic of the P700 oxidation and reduction state. The rate of decline in the kinetic curve of the dark re-reduction of P700^+^ after turning off the FR light (i.e., the rate of reduction of P700) indicates the rate of cyclic electron transport, and the half-time (*t*_1/2_) of the dark decay of the P700^+^ signal is usually used to estimate the rate of reduction of P700 (Popova et al., 2018; Gerganova et al., 2019).

### Measurement of Fluorescence at Dawn

At 3:00 a.m., fluorescence parameters were collected using SP analysis and the P700^+^ Fluo mode of the Dual-PAM-100 at 0 μmol m^–2^ s^–1^ light intensity. In other words, the minimal fluorescence from dark-adapted leaf (*F*_0_), maximal fluorescence from dark-adapted leaf (*F*_m_), the maximum quantum efficiency of PSII photochemistry (*F*_v_/*F*_m_), and the P700 maximum fluorescence signal under dark adaptation (*P*_m_) were measured after dark adaptation.

### Measurement of Photosynthetic Pigment Content

Three different light-demanding plants, after adequate dark adaptation, were suddenly exposed to simulated sunflecks for 30 min with a light intensity of 50, 100, 400, 800, and 1,600 μmol m^–2^ s^–1^, respectively, followed by dark treatment for 30 min, and the whole process lasted for 60 min. The leaves were collected at 0, 10, 20, 30, 45, and 60 min, respectively. Leaves were wrapped in tin foil and quickly stored in liquid nitrogen. Later, the leaves were stored in a refrigerator at −80°C. According to the method described by Susan and Björkman (1990) with minor modifications, the content of violaxanthin (V), anteraxantin (A), and zeaxanthin (Z) were determined by high-performance liquid chromatography (HPLC, Agilent 1260, United States). The xanthophylls de-epoxidation state is DES = (V + A)/(V + A + Z).

### Statistical Analysis

Statistics were performed using Microsoft Excel 2010 software, and analyses were performed using SPSS19.0 software. The results represent mean values derived from five independent experiments. One-way ANOVA with an α = 0.05 significance level was used to determine whether there are significant differences between different treatments. The graph data were mean ± SE and were plotted using the GraphPad Prism 8.3.0 software.

## Results

### Effects of Transient Light on PSII Activity in Different Light-Demanding Plants

**FIGURE 1:**
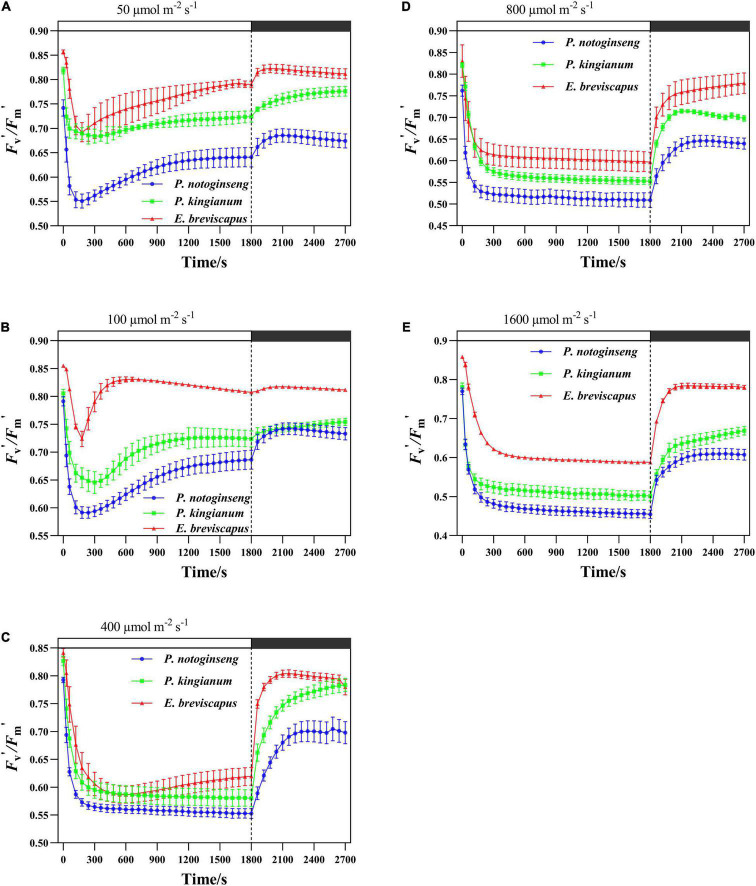
Effects of transient light on PSII maximum efficiency (*F*_v_′/*F*_m_′) in different light-demanding plants. The *Panax notoginseng* (indicated in blue) and *Polygonatum kingianum* (indicated in green) and *Erigeron breviscapus* (indicated in red) were induced for 30 min in simulated dynamic light of 50 μmol m^−2^ s^−1^ **(A)**, 100 μmol m^−2^ s^−1^ **(B)**, 400 μmol m^−2^ s^−1^ **(C)**, 800 μmol m^−2^ s^−1^ **(D)** and 1600 μmol m^−2^ s^−1^ **(E)** respectively (the left of the dotted line), and then dark recovery was conducted for 15 min (the right of the dotted line), the value is the means ± SE. (*n* = 5; the same below).

Different light-demanding plants showed a significant difference in PSII activity under simulated dynamic light (Figure 1). The *F*_v_′/*F*_m_′ of *P. notoginseng*, *P. kingianum*, and *E. breviscapus* exposed to simulated dynamic light decreased rapidly. The decreased degree of *F*_v_′/*F*_m_′ in three different light-demanding plants increased as light intensity increased. *F*_v_′/*F*_m_′ recovered to a certain level after a drop in dynamic low light (50 and 100 μmol m^–2^ s^–1^), while there was no significant recovery after a drop in dynamic high light (400, 800, and 1,600 μmol m^–2^ s^–1^). During the dark recovery period, the recovery level of *F*_v_′/*F*_m_′ decreased with the increase in light intensity. These results suggest that the PSII activity reduced when three different light-demanding plants were exposed to simulated dynamic light, especially under dynamic high light.

When exposed to simulated dynamic light, *F*_v_′/*F*_m_′ declined in descending order for the sun plant *E. breviscapus*, the intermediate plant *P. kingianum*, to the shade plant *P. notoginseng* (Figure 1). Differences in *F*_v_′/*F*_m_′ induction-related traits (T_30_, T_50_, T_90_, IS_300S_, IS_600S_, and IS_1200S_) in different light-demanding plants were found in this study (Table 1). Compared with the shade plant *P. notoginseng*, the T_30_, T_50_, T_90_, IS_300S_, IS_600S_, and IS_1200S_ of sun plant *E. breviscapus* were significantly elevated. In addition, *E. breviscapus* recovered to a greater extent under dynamic low light (Figure 1). During the dark recovery period, the recovery degree of *E. breviscapus* was the highest, followed by *P. kingianum* and finally by *P. notoginseng*. This suggests that the PSII activity of sun plants was higher than that of shade plants when dark-adapted plants are exposed to transient light.

**TABLE 1:** Parameters of the PSII maximum efficiency (*F*_v_′/*F*_m_′) induction properties in *P. notoginseng* and *P. kingianum* and *E. breviscapus* studied.

Light intensity	Species	T_30_(S)	T_50_(S)	T_90_(S)	IS_300S_	IS_600S_	IS_1200S_
50	*P. notoginseng*	54.591 ± 2.156 c	34.450 ± 1.533 c	7.600 ± 0.651 c	0.562 ± 0.021 b	0.595 ± 0.021 b	0.634 ± 0.028 b
	*P. kingianum*	81.325 ± 2.607 b	52.485 ± 2.184 b	13.658 ± 0.661 b	0.669 ± 0.019 a	0.697 ± 0.007 a	0.732 ± 0.007 a
	*E. breviscapus*	118.638 ± 2.336 a	90.564 ± 3.739 a	28.161 ± 1.520 a	0.711 ± 0.032 a	0.739 ± 0.032 a	0.773 ± 0.017 a
100	*P. notoginseng*	50.824 ± 1.333 c	30.370 ± 0.902 c	6.290 ± 0.414 b	0.594 ± 0.010 c	0.624 ± 0.011 c	0.674 ± 0.015 c
	*P. kingianum*	72.886 ± 2.127 b	42.755 ± 1.087 b	7.215 ± 0.590 b	0.666 ± 0.010 b	0.712 ± 0.016 b	0.743 ± 0.010 b
	*E. breviscapus*	110.704 ± 3.216 a	82.259 ± 2.586 a	28.298 ± 1.243 a	0.804 ± 0.007 a	0.832 ± 0.003 a	0.821 ± 0.002 a
400	*P. notoginseng*	69.108 ± 4.069 c	39.977 ± 2.686 b	7.278 ± 1.251 b	0.564 ± 0.007 a	0.560 ± 0.007 a	0.556 ± 0.008 b
	*P. kingianum*	90.283 ± 3.078 b	50.504 ± 1.648 b	7.210 ± 0.481 b	0.595 ± 0.015 a	0.587 ± 0.015 a	0.583 ± 0.015 ab
	*E. breviscapus*	146.472 ± 3.464 a	259.012 ± 164.894 a	30.643 ± 0.981 a	0.606 ± 0.021 a	0.587 ± 0.015 a	0.605 ± 0.018 a
800	*P. notoginseng*	52.635 ± 1.346 c	30.583 ± 0.751 c	5.006 ± 0.232 b	0.523 ± 0.016 b	0.517 ± 0.016 b	0.512 ± 0.016 b
	*P. kingianum*	85.592 ± 3.090 b	47.819 ± 1.854 b	6.372 ± 0.343 b	0.614 ± 0.009 ab	0.608 ± 0.009 ab	0.603 ± 0.009 ab
	*E. breviscapus*	134.591 ± 4.179 a	80.716 ± 3.065 a	19.614 ± 1.865 a	0.575 ± 0.024 a	0.563 ± 0.024 a	0.556 ± 0.023 a
1600	*P. notoginseng*	62.923 ± 5.102 c	35.838 ± 2.981 c	5.977 ± 0.754 b	0.524 ± 0.011 b	0.516 ± 0.010 b	0.507 ± 0.010 b
	*P. kingianum*	85.300 ± 4.281 b	47.626 ± 1.973 b	6.024 ± 0.641 b	0.481 ± 0.045 b	0.469 ± 0.045 b	0.460 ± 0.044 b
	*E. breviscapus*	177.801 ± 5.399 a	105.835 ± 3.780 a	24.588 ± 1.457 a	0.621 ± 0.003 a	0.600 ± 0.003 a	0.592 ± 0.003 a

*The data in the table are mean ± SE (n = 5). Different letters indicate significant differences between materials (P < 0.05). T_30_(S), the time required to reach 30% of maximum F_v_′/F_m_′; T_50_(S), the time required to reach 50% of maximum F_v_′/F_m_′; T_90_(S), the time required to reach 90% of maximum F_v_′/F_m_′; IS_300S_, the induction state of different light-demanding plants after 300 s light; IS_600S_, the induction state of different light-demanding plants after 600 s light; IS_1200S_, the induction state of different light-demanding plants after 1,200 s light.*

### Changes of Energy Quenching in Different Light-Demanding Plants Under Transient Light

**FIGURE 2:**
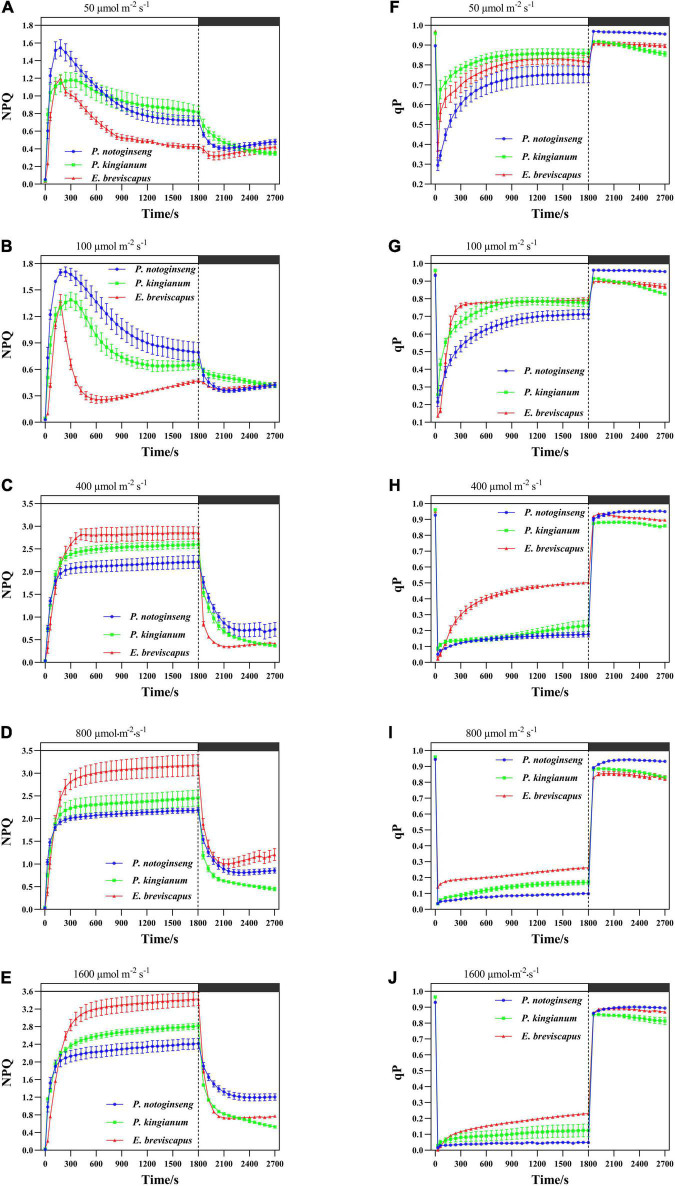
Effects of transient light on non-photochemical quenching (NPQ) **(A–E)** and photochemical quenching (qP) **(F–J)** in different light-demanding plants.

Non-photochemical quenching of different light-demanding plants all rose rapidly when plants were exposed to different transient dynamic lights (Figures 2A–E). The results showed that *P. notoginseng*, *P. kingianum*, and *E. breviscapus* exposed to transient, dynamic high light will dissipate energy mainly through the NPQ pathway. When three different light-demanding plants were exposed to transient low light conditions, NPQ increased rapidly to dissipate more energy at first but decreased gradually with the increase in qP at the later stage. During the dark recovery period, qP recovered rapidly under transient light, while NPQ remained at high levels under transient high light. The slight increase in NPQ under dark conditions may be related to the shorter measurement period. These results indicate that when the three different light-demanding plants were suddenly exposed to transient light, energy dissipation was *via* qP and NPQ in low light. In contrast, energy dissipation was mainly *via* NPQ in high light.

The NPQ of the sun plant *E. breviscapus* rose slower under transient dynamic light, and the increased amplitude was smaller under low light, while the increased amplitude was larger under high light (Figures 2A–E). Conversely, the NPQ of shade plant *P. notoginseng* increased faster under transient dynamic light and had a larger value under low light but a smaller value under high light (Figure 2). The intermediate plant *P. kingianum* was located in between *P. notoginseng* and *E. breviscapus* (Figure 2). In contrast, sun plant *E. breviscapus* exhibited significantly higher values of T_30_, T_50_, T_90_, IS_300S_, IS_600S_, and IS_1200S_ compared with shade plant *P. notoginseng* (Table 2). During the dark recovery period, the recovery degree of *E. breviscapus* was the highest, followed by *P. kingianum* and finally by *P. notoginseng*. The recovery degree of *E. breviscapus* was close to the initial value at all light intensities, while that of *P. notoginseng* and *P. kingianum* was lower. In short, the NPQ initiated by the shade plant was larger in low light, while that of the sun plant was weaker at this time. However, it was the opposite under high light. This showed that the sun plant had a better photoprotective ability, while the shade plant was more sensitive to transient dynamic light changes.

**TABLE 2:** Parameters of the non-photochemical quenching (NPQ) induction properties in *P. notoginseng* and *P. kingianum* and *E. breviscapus* studied.

Light intensity	Species	T_30_(S)	T_50_(S)	T_90_(S)	IS_300S_	IS_600S_	IS_1200S_
50	*P. notoginseng*	25.244 ± 1.434 b	48.923 ± 2.478 b	132.264 ± 7.702 b	1.421 ± 0.184 b	1.105 ± 0.141 b	0.772 ± 0.112 a
	*P. kingianum*	20.232 ± 0.458 c	41.969 ± 1.534 b	134.843 ± 12.926 b	1.180 ± 0.183 b	1.047 ± 0.102 b	0.887 ± 0.192 a
	*E. breviscapus*	54.804 ± 2.321 a	92.195 ± 2.840 a	177.870 ± 8.322 a	2.213 ± 0.113 a	1.869 ± 0.207 a	1.137 ± 0.125 a
100	*P. notoginseng*	25.750 ± 2.550 b	51.177 ± 4.724 b	150.935 ± 11.494 a	1.676 ± 0.070 a	1.364 ± 0.114 a	0.900 ± 0.109 a
	*P. kingianum*	22.663 ± 4.051 b	45.993 ± 7.906 b	162.940 ± 19.063 a	1.448 ± 0.107 ab	0.725 ± 0.096 b	0.448 ± 0.013 b
	*E. breviscapus*	51.221 ± 2.602 a	85.553 ± 4.359 a	154.668 ± 7.941 a	1.180 ± 0.090 b	0.503 ± 0.037 b	0.361 ± 0.016 b
400	*P. notoginseng*	16.251 ± 1.431 c	37.491 ± 3.317 c	292.544 ± 28.944 a	2.064 ± 0.115 c	2.114 ± 0.124 c	2.173 ± 0.135 c
	*P. kingianum*	21.754 ± 0.891 b	49.721 ± 2.017 b	349.009 ± 15.753 a	2.342 ± 0.057 b	2.473 ± 0.065 b	2.560 ± 0.081 b
	*E. breviscapus*	31.617 ± 1.632 a	72.608 ± 3.139 a	299.924 ± 5.779 a	2.726 ± 0.043 a	2.955 ± 0.080 a	3.074 ± 0.078 a
800	*P. notoginseng*	13.750 ± 0.837 c	31.969 ± 1.941 c	275.164 ± 18.055 c	2.007 ± 0.055 b	2.072 ± 0.058 b	2.140 ± 0.055 b
	*P. kingianum*	19.951 ± 0.889 b	46.144 ± 2.062 b	371.379 ± 21.118 b	2.228 ± 0.177 b	2.306 ± 0.178 b	2.379 ± 0.177 b
	*E. breviscapus*	34.806 ± 1.034 a	79.003 ± 2.722 a	479.641 ± 12.339 a	3.237 ± 0.121 a	3.468 ± 0.123 a	3.564 ± 0.145 a
1600	*P. notoginseng*	17.796 ± 2.184 c	41.502 ± 5.085 c	372.313 ± 45.298 b	2.190 ± 0.090 b	2.261 ± 0.102 c	2.362 ± 0.113 c
	*P. kingianum*	25.428 ± 1.251 b	60.650 ± 4.777 b	501.392 ± 27.202 a	2.486 ± 0.084 ab	2.683 ± 0.090 b	2.851 ± 0.073 b
	*E. breviscapus*	49.484 ± 1.267 a	109.465 ± 2.890 a	590.046 ± 10.902 a	2.794 ± 0.125 a	3.228 ± 0.177 a	3.350 ± 0.168 a

*The data in the table are mean ± SE (n = 5). Different letters indicate significant differences between materials (P < 0.05). T_30_(S), the time required to reach 30% of maximum NPQ; T_50_(S), the time required to reach 50% of maximum NPQ; T_90_(S), the time required to reach 90% of maximum NPQ; IS_300S_, the induction state of different light-demanding plants after 300 s light; IS_600S_, the induction state of different light-demanding plants after 600 s light; IS_1200S_, the induction state of different light-demanding plants after 1,200 s light.*

### Energy Distribution of PSII in Different Light-Demanding Plants Under Transient Light

**FIGURE 3:**
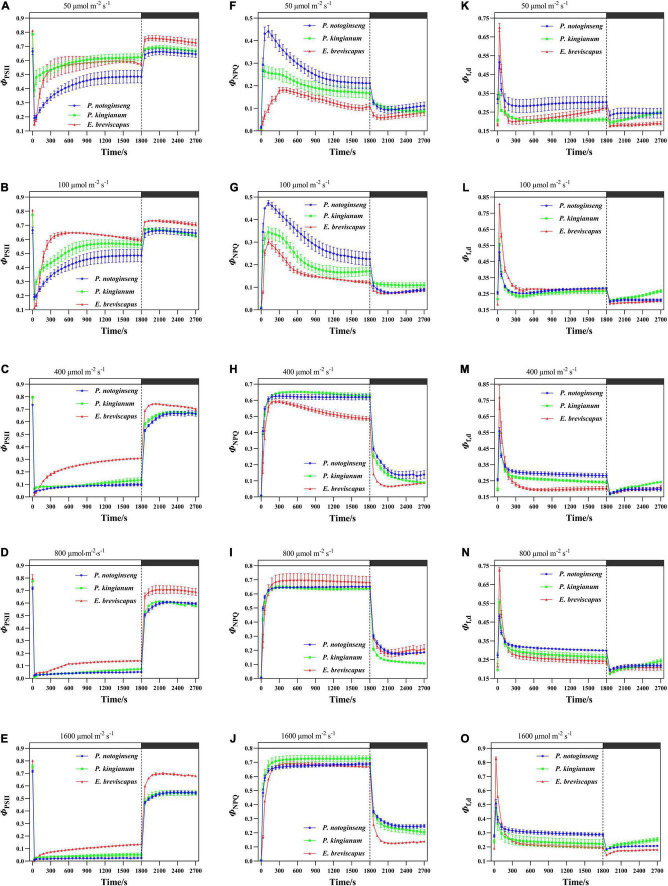
Effects of transient light on the quantum yield of PSII photochemistry (Φ_PSII_) **(A–E)** and quantum yield of ΔpH, xanthophyll-regulated thermal energy dissipation (Φ_NPQ_) **(F–J)**, and constitutive thermal dissipation and fluorescence (Φ_f,d_) **(K–O)** in different light-demanding plants.

The light energy absorbed by PSII of *P. notoginseng*, *P. kingianum*, and *E. breviscapus* was mainly consumed through the photochemical reaction pathway (Figures 3A,B) when exposed to transient low light (50 and 100), while the remaining energy was consumed through the NPQ pathway (Figures 3F,G) and the fluorescence dissipation pathway (Figures 3K,L). Φ_PSII_ decreased with the increase in transient light intensity when plants changed from a dark environment to transient light (Figures 3A–E). The proportion of Φ_PSII_ was smaller (Figures 3C–E) under high light (400, 800, and 1,600 μmol m^–2^ s^–1^), while the proportion of Φ_NPQ_ increased gradually with the increase in simulated light intensity (Figures 3H–J). Φ_f,d_ remained relatively stable under transient light conditions. In addition, Φ_PSII_ and Φ_NPQ_ could not be restored to their initial values when *P. notoginseng*, *P. kingianum*, and *E. breviscapus* changed from simulated light to darkness, especially under transient high light conditions (Figure 3). These results suggest that plants dissipate energy mainly through Φ_PSII_ under low light, and the proportion of Φ_NPQ_ and Φ_f,d_ was also greater. The Φ_NPQ_ was significantly higher under high light.

The Φ_PSII_ of the sun plant *E. breviscapus* was higher than shade plant *P. notoginseng* at transient light conditions, while Φ_f,d_ was slightly greater for the shade plant. However, the Φ_NPQ_ of the shade plant was higher under low light, while it was greater for the sun plant in high light. In addition, Φ_PSII_, Φ_NPQ_, and Φ_f,d_ of the sun plant increased slower. The Φ_NPQ_ was lower than Φ_f,d_ in the sun plant *E. breviscapus* under low light, while the Φ_NPQ_ of the shade plant *P. notoginseng* was close to Φ_f,d_. These results indicate that although the light energy distribution of different light-demanding plants was similar in general, the photochemical efficiency of sun plants was relatively greater, and they were more insensitive to changes in a transient dynamic light. While shade plants were more sensitive to transient dynamic light changes, the fluorescence dissipation was larger.

### Changes of PSI Parameters in Different Light-Demanding Plants Under Transient Light

**FIGURE 4:**
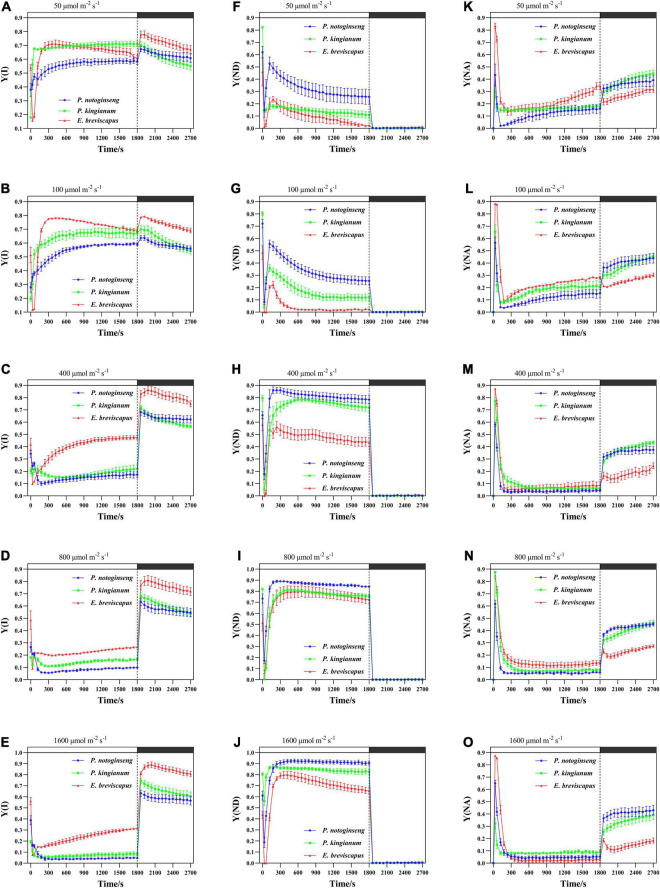
Effects of transient light on the quantum yield of photochemical energy conversion [Y(I)] **(A–E)** and quantum yield of non-photochemical energy dissipation due to donor side limitation [Y(ND)] **(F–J)** and quantum yield of non-photochemical energy dissipation due to acceptor side limitation [Y(NA)] **(K–O)** in different light-demanding plants.

**FIGURE 5:**
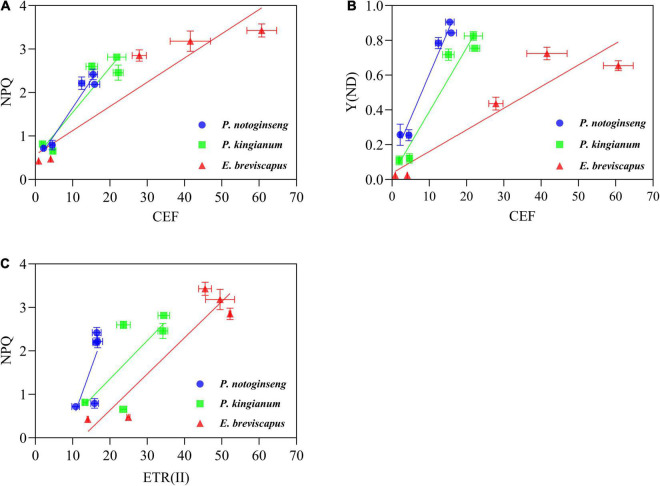
Correlation between CEF and NPQ **(A)**, CEF and Y(ND) **(B)**, ETR(II) and NPQ **(C)** under different transient light intensity (Correlation was performed on the points taken after 30 min of illumination).

The light energy absorbed by the PSI was mainly distributed to the quantum yield of photochemical energy conversion [Y(I)] (Figures 4A,B) when exposed to transient low light (50 and 100 μmol m^–2^ s^–1^). However, the light energy was mainly distributed to Y(ND) (Figures 4H–J) under transient high light. At the same time, the ΔpH generated by CEF will strengthen Y(ND), so Y(ND) has a positive correlation with CEF (Figure 5). In addition, the proportion of quantum yield of non-photochemical energy dissipation due to acceptor side limitation [Y(NA)] (Figures 4K–O) was low at transient light conditions. For the different light-demanding plants, Y(I) of the shade plant *P. notoginseng* was lower than that of the sun plant *E. breviscapus* at all light intensities, while Y(ND) held the opposite. In addition, the response to transient light was faster in shade plants, and Y(NA) was slightly higher in sun plants. These results suggest that sun plants have relatively more photochemical efficiency of PSI and are more retarded to changes in a transient dynamic light. Shade plants were more sensitive to changes in transient dynamic light and were more efficient at dissipating heat at the donor terminal.

### Effects of Transient Light on Electron Transport Rate in Different Light-Demanding Plants

**FIGURE 6:**
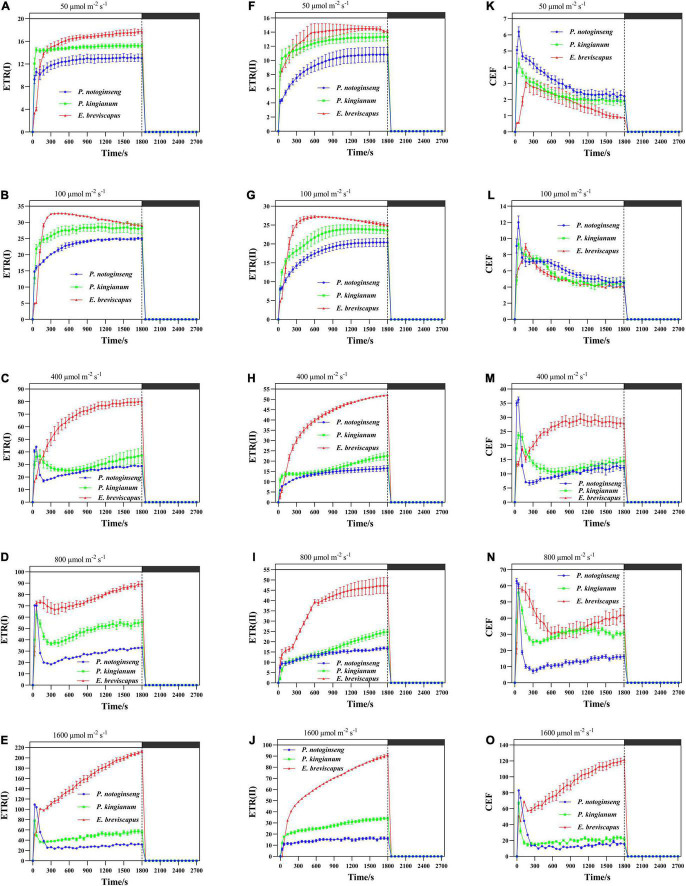
Effects of transient light on electron transport rate in PSI [ETR(I)] **(A–E)** and electron transport rate in PSII [ETR(II)] **(F–J)** and cyclic electron transport (CEF) **(K–O)** in different light-demanding plants.

The ETR(II), ETR(I), and CEF increased rapidly when plants were exposed to transient light (Figure 6). The ETR(I) and ETR(II) of sun plants were higher than shade plants at all transient light levels (especially at high light) but rose more slowly (Figures 6A–J). CEF was higher in shade plants in low light, while it was significantly higher and rose more slowly in sun plants in high light. Sun plant *E. breviscapus* exhibited significantly higher values of T_30_, T_50_, T_90_, IS_300S_, IS_600S_, and IS_1200S_ than shade plant *P. notoginseng* (Table 3). This indicates that high CEF was stimulated by transient light to protect the photosystem from damage, and shade plants were more sensitive to transient dynamic light changes. Meanwhile, CEF and ETR(II) were positively correlated with NPQ, and the correlation was highest for shade plants, followed by intermediate plants, and lowest for sun plants (Figure 5). The correlation between ETR(II) and NPQ was higher than that between CEF and NPQ.

**TABLE 3:** Parameters of the cyclic electron flow (CEF) induction properties in *P. notoginseng* and *P. kingianum* and *E. breviscapus* studied.

Light intensity	Species	T_30_(S)	T_50_(S)	T_90_(S)	IS_300S_	IS_600S_	IS_1200S_
50	*P. notoginseng*	7.676 ± 0.546 b	17.150 ± 0.812 b	67.674 ± 0.416 b	4.020 ± 0.196 a	3.380 ± 0.128 a	2.520 ± 0.102 a
	*P. kingianum*	5.135 ± 0.367 b	10.968 ± 0.478 c	71.925 ± 1.071 b	3.480 ± 0.146 b	2.820 ± 0.073 b	2.120 ± 0.153 b
	*E. breviscapus*	46.453 ± 1.288 a	77.422 ± 2.146 a	135.559 ± 3.947 a	2.320 ± 0.124 c	1.580 ± 0.128 c	1.040 ± 0.093 c
100	*P. notoginseng*	6.733 ± 0.548 c	13.728 ± 0.939 c	53.766 ± 1.722 c	7.040 ± 0.140 a	6.920 ± 0.150 a	6.360 ± 0.172 a
	*P. kingianum*	11.561 ± 0.730 b	22.571 ± 1.222 b	63.560 ± 1.697 b	7.780 ± 0.262 a	6.740 ± 0.262 a	4.880 ± 0.242 b
	*E. breviscapus*	59.390 ± 1.718 a	98.783 ± 1.901 a	176.769 ± 4.232 a	7.060 ± 0.343 a	5.600 ± 0.167 b	4.480 ± 0.136 b
400	*P. notoginseng*	5.192 ± 0.172 b	10.486 ± 0.413 c	29.296 ± 1.211 c	5.920 ± 0.107 c	6.840 ± 0.204 c	10.420 ± 0.599 b
	*P. kingianum*	6.357 ± 0.443 b	13.126 ± 0.824 b	52.286 ± 2.008 b	11.700 ± 1.233 b	9.800 ± 0.445 b	11.400 ± 0.661 b
	*E. breviscapus*	19.705 ± 0.641 a	36.905 ± 0.500 a	105.052 ± 3.298 a	17.940 ± 0.783 a	27.340 ± 0.787 a	31.020 ± 1.360 a
800	*P. notoginseng*	5.129 ± 0.255 c	7.377 ± 0.422 c	33.122 ± 2.784 c	6.060 ± 0.586 c	8.960 ± 0.571 b	11.580 ± 0.731 c
	*P. kingianum*	9.196 ± 0.331 a	19.344 ± 0.718 a	78.431 ± 1.831 a	17.420 ± 1.391 b	18.560 ± 0.539 a	22.620 ± 1.273 b
	*E. breviscapus*	7.826 ± 0.307 b	14.955 ± 0.398 b	58.349 ± 2.764 b	23.740 ± 1.429 a	20.180 ± 0.862 a	30.300 ± 1.455 a
1600	*P. notoginseng*	1.922 ± 0.116 b	5.192 ± 0.126 c	20.327 ± 1.352 b	17.980 ± 1.565 b	14.120 ± 1.012 c	16.140 ± 1.148 b
	*P. kingianum*	2.676 ± 0.184 b	6.146 ± 0.269 b	35.087 ± 1.797 a	20.220 ± 1.399 b	20.320 ± 1.693 b	20.540 ± 1.869 b
	*E. breviscapus*	5.741 ± 0.487 a	8.331 ± 0.290 a	24.894 ± 1.795 b	31.000 ± 1.404 a	35.820 ± 2.038 a	51.580 ± 1.306 a

*The data in the table are mean ± SE (n = 5). Different letters indicate significant differences between materials (P < 0.05). T_30_(S), the time required to reach 30% of maximum CEF; T_50_(S), the time required to reach 50% of maximum CEF; T_90_(S), the time required to reach 90% of maximum CEF; IS_300S_, the induction state of different light-demanding plants after 300 s light; IS_600S_, the induction state of different light-demanding plants after 600 s light; IS_1200S_, the induction state of different light-demanding plants after 1,200 s light.*

**FIGURE 7:**
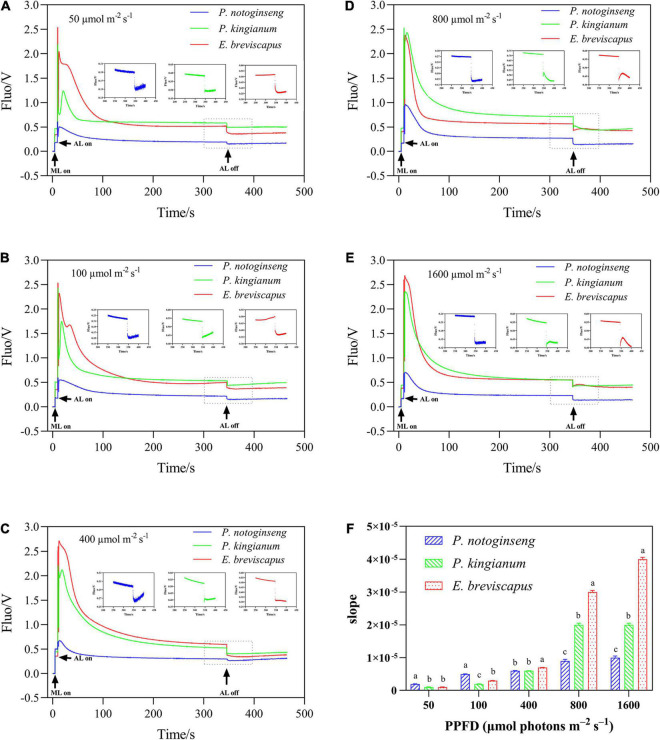
Effects of different plants in different light intensities on post-illumination. The *P. notoginseng* (indicated in blue) and *P. kingianum* (indicated in green) and *E. breviscapus* (indicated in red) was measured in simulated dynamic light of 50 μmol m^−2^ s^−1^ **(A)**, 100 μmol m^−2^ s^−1^ **(B)**, 400 μmol m^−2^ s^−1^ **(C)**, 800 μmol m^−2^ s^−1^ **(D)** and 1600 μmol m^−2^ s^−1^ **(E)** respectively. Arrows indicate the timing of on and off points of measuring light (ML), actinic light (AL). Insets are magnified traces from the boxed area of transient increase. Panel **(F)** is the slope of Post-illumination.

The chlorophyll fluorescence reached a maximum when the dark-adapted plants were suddenly exposed to transient light and then gradually decreased and stabilized. In contrast, chlorophyll fluorescence increased transiently after turning off photochemical light (AL), which was thought to be an NDH-mediated CEF around PSI in higher plants (Sun et al., 2017; Figures 7A–E). NDH-mediated CEF increased gradually with the increase in light intensity. The CEF of shade plant *P. notoginseng* was significantly higher under low light, while the CEF of sun plant *E. breviscapus* was higher under high light conditions (Figure 7F). These results suggest that NDH-mediated CEF and CEF differed significantly among different light-demanding plants, and the CEF was higher in the sun plant under high light conditions.

### Differences in the Redox Kinetics of P700 in Different Light-Demanding Plants

**FIGURE 8:**
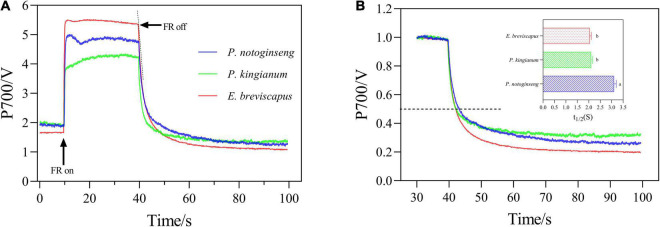
P700^+^ re-reduction curves of the *P. notoginseng* (indicated in blue), *P. kingianum* (indicated in green), and *E. breviscapus* (indicated in red). Arrows indicate the timing of on and off points of far-red light (FR). **(A)** P700^+^ Re-reduction curves. **(B)** Enlarged display of normalized P700 signal after FR light was removed.

**FIGURE 9:**
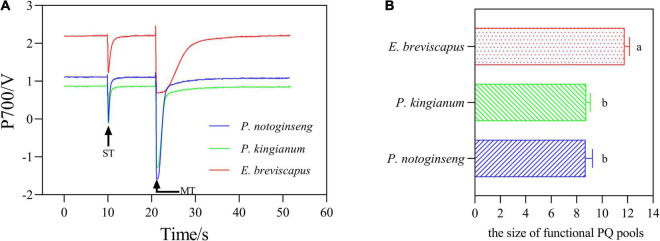
Photochemical quenching (PQ) pool curves of the *P. notoginseng* (indicated in blue), *P. kingianum* (indicated in green), and *E. breviscapus* (indicated in red). The P700 signal was determined during single turnover flashes (ST, 50 ms, PQ pool being oxidized) followed by multiple turnover flashes (MT, 50 ms, PQ pool is fully reduced). **(A)** PQ pool curves. **(B)** The ratio of MT area/ST area is used to estimate the size of functional PQ pools.

The transition of the P700 signal from the maximum oxidation state to the reduced state was analyzed after the FR was turned off (Figure 8). The slope of the curve (which can be expressed by the size of the half-life) indicates the rate of CEF and reflects the ability of cyclic electron transport. As shown in figure, the half-life of *P. notoginseng* was significantly larger than that of *P. kingianum* and *E. breviscapus*, indicating that shade plant *P. notoginseng* had a lower capacity for cyclic electron transport. The PQ pool of shade plant *P. notoginseng* was smaller, while the PQ pool of sun plant *E. breviscapus* was significantly higher (Figure 9). These results suggest that shade plants had a smaller PQ pool, and electron transport is easy to saturate, which hinders electron transport and results in a lower CEF in high light.

### Differences in Chlorophyll Fluorescence Parameters at the Dawn of Light-Demanding Plants

**FIGURE 10:**
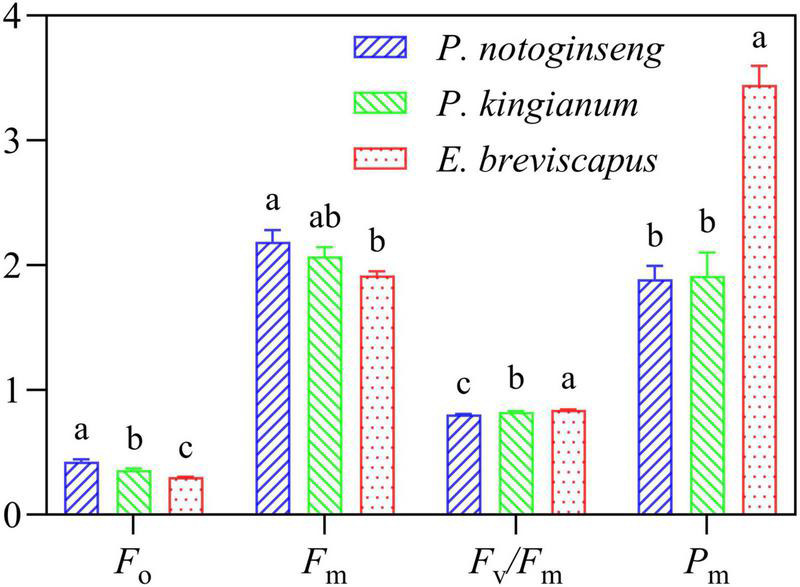
Chlorophyll fluorescence parameters at the dawn of *P. notoginseng* (indicated in blue), *P. kingianum* (indicated in green), and *E. breviscapus* (indicated in red). Different letters indicate significant differences (*P* < 0.05).

The minimal fluorescence from dark-adapted leaf (*F*_0_) and maximal fluorescence from dark-adapted leaf (*F*_m_) in the shade plant *P. notoginseng* were significantly higher. However, the maximum quantum efficiency of PSII photochemistry (*F*_v_/*F*_m_) and P700 maximum fluorescence signal under dark adaptation (*P*_m_) were significantly lower than the sun plant *E. breviscapus* (Figure 10). A higher *P*_m_ indicates that the amplitude of the P700^+^ of the sun plant is higher than the shade plant. These results suggest that the activities of PSI and PSII were lower in shade plants, and this may be a reason why the photosystem was easily inhibited under high light.

### Changes of De-Epoxidation State in Different Light-Demanding Plants Under Transient Light

**FIGURE 11:**
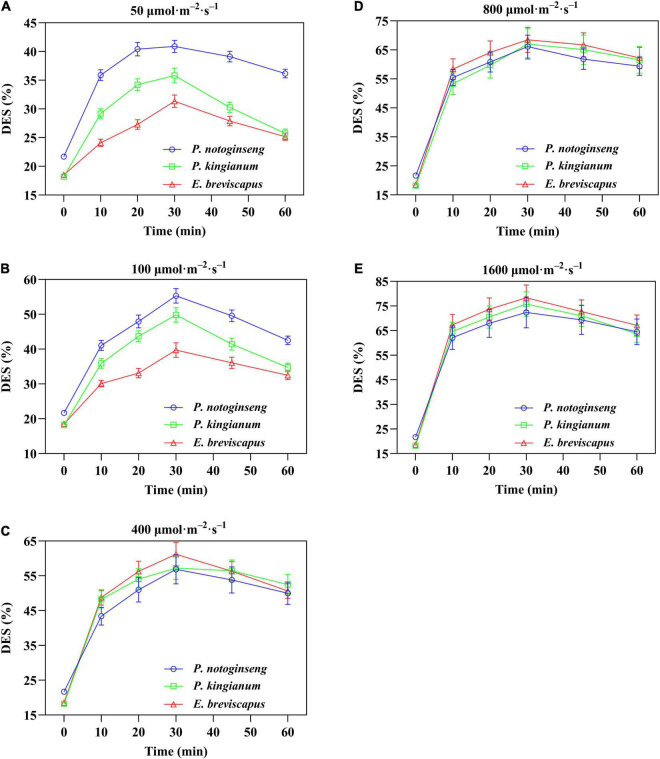
Effects of transient light on xanthophylls de-epoxidation state (DES) (V+A/V+A+Z) in different light-demanding plants. The *P. notoginseng* (blue) and *P. kingianum* (green) and *E. breviscapus* (red) were induced for 30 min in dynamic light of 50 μmol m^−2^ s^−1^ **(A)**, 100 μmol m^−2^ s^−1^ **(B)**, 400 μmol m^−2^ s^−1^ **(C)**, 800 μmol m^−2^ s^−1^ **(D)** and 1600 μmol m^−2^ s^−1^ **(E)** respectively, and then dark recovery was conducted for 30 min.

**FIGURE 12:**
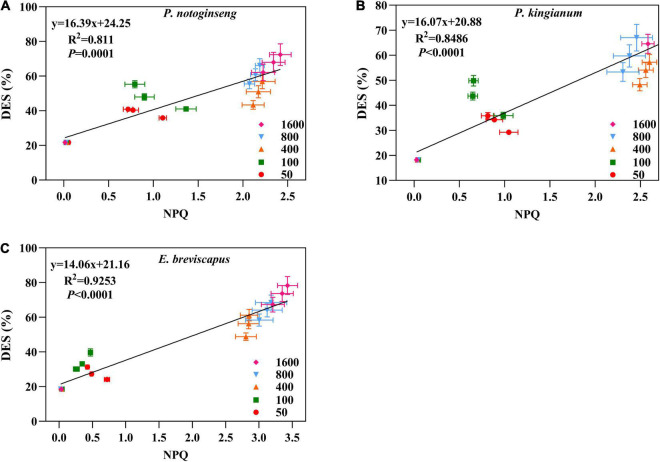
Correlation between NPQ and xanthophylls de-epoxidation state (DES) in *P. notoginseng* **(A)** and *P. kingianum* **(B)** and *E. breviscapus* **(C)**.

The de-epoxidation state of different light-demanding plants increased rapidly when suddenly exposed to light, gradually increased with the increase of light intensity, and then showed a downward trend after turning to darkness (Figure 11). In addition, the DES of shade plants was significantly higher than the other two plants under low light (50 and 100 μmol m^–2^ s^–1^). Under high light (400, 800, and 1,600 μmol m^–2^ s^–1^), the DES of the sun plants was higher. This is consistent with the change in NPQ. Meanwhile, DES was positively correlated with NPQ, and the correlation was highest for sun plants, followed by intermediate plants, and lowest for shade plants (Figure 12). These results indicated that the sun plants initiated a greater lutein cycle under high light and had greater photoprotection.

## Discussion

### Sun Plants Show Greater Photochemical Efficiency While Shade Plants Are More Sensitive to Dynamic Light

Non-photochemical quenching is the most effective first line of defense for plants to dissipate excess light energy and to exert photoprotection (Maxwell and Johnson, 2000; Demmig-Adams et al., 2012; Wei et al., 2020). Sun plant *Magnolia grandiflora* possesses greater NPQ in high light, which effectively protects photosynthetic apparatus from photodamage (Valladares et al., 2002). Although shade plant *Psychotria asiatica* also exhibits relatively higher NPQ under high light, PSI and PSII are still severely photoinhibited (Huang et al., 2015). NPQ and Φ_NPQ_ increased rapidly when different light-demanding plants were exposed to simulated dynamic light (Figures 2A–E, 3F–J), which is consistent with the results suggested by Tausz et al. (2005) and Zhang et al. (2021) that NPQ plays an important protective role in plants exposed to transient light conditions. A larger NPQ in low light (50 and 100 μmol m^–2^ s^–1^), accompanied by a lower qP (Figures 2A,B,F,G), indicated that declined *F*_v_′/*F*_m_′ of the shade plant *P. notoginseng* results in low photochemical efficiency under transient low light conditions (Figures 1A,B). Both NPQ and qP were larger in the sun plants under high light (400, 800, and 1,600 μmol m^–2^ s^–1^) (Figures 2C–E,H–J). In addition, the change in NPQ is consistent with the change in DES, and NPQ is positively correlated with DES under simulated dynamic light (Figure 12). These results suggest that the V cycle is activated in different light-demanding plants and that it is positively correlated with NPQ. Meanwhile, *F*_v_′/*F*_m_′ was strongly inhibited in different light-demanding plants, but sun plants still maintained a relatively higher *F*_v_′/*F*_m_′ (Figures 1C–E), suggesting that the photochemical efficiency and photoprotective capacity of the sun plants were greater under transient high light. The NPQ of shade plants increased faster than the others under transient light conditions, and it takes less time to reach the maximum value (Figures 2A–E and Table 2), which may be related to the PsbS as suggested by Johnson and Ruban (2010, 2011). This indicates that the NPQ of shade plants is more sensitive to changes in light intensity. Thus, sun plants show greater photochemical efficiency and photoprotective capacity in high light conditions, contributed by higher photochemical quantum efficiency of PSII and NPQ, while the NPQ of shade plants is more sensitive to changes in a dynamic light.

The NPQ of different light-demanding plants decreased obviously after the transition from light to darkness, whereas *F*_v_′/*F*_m_′ and qP increased significantly (Figures 1, 2). *F*_v_′/*F*_m_′ and NPQ hardly returned to the initial state, especially in transient high light conditions. A possible explanation is that the relaxation rate of NPQ lags behind the induction rate and is exacerbated by long-term exposure to high light conditions (Pérez-Bueno et al., 2008) so that NPQ inhibits photosynthetic quantum yield after the light-dark transition (Murchie and Niyogi, 2011). The recovery of *F*_v_′/*F*_m_′ and NPQ was closer to the initial value in the sun plant *E. breviscapus* than the shade plant *P. notoginseng*. This may be due to the fact that sun plants exhibit a higher PSII activity and a greater photoprotection under dynamic light conditions (Sato et al., 2014), resulting in a fast recovery of *F*_v_′/*F*_m_′ and NPQ.

### More Light Energy Is Allocated to Φ_PSII_ in Sun Plants, While Relatively More Light Energy Is Allocated to Φ_f,d_ in Shade Plants

It has been commonly accepted that light energy absorbed by plants is allocated into a quantum yield of ΔpH and xanthophyll-regulated thermal energy dissipation (Φ_NPQ_), the quantum yield of PSII photochemistry (Φ_PSII_), and constitutive thermal dissipation and fluorescence (Φ_f,d_). In sun plant grape (*V. vinifera*) and rice (*O. sativa*), Φ_PSII_ and Φ_f,d_ are smaller and Φ_NPQ_ is larger under high light conditions, suggesting that most of the light energy absorbed by plants under high light conditions is safely dissipated in the form of NPQ (Hendrickson et al., 2004; Ishida et al., 2014). This is consistent with the result obtained in this study that a larger Φ_NPQ_ was observed in *P. notoginseng*, *P. kingianum*, and *E. breviscapus* (Figure 3). Notably, Φ_PSII_ was larger in sun plants than shade plants when they were exposed to transient light (Figures 3A–E), and this may be related to the photochemical efficiency. *F*_v_′/*F*_m_′ was higher in sun plants when exposed to dynamic light (Figure 1), indicating that the captured light energy is more used for the formation of photochemical reduction force to contribute higher Φ_PSII_. Furthermore, Φ_f,d_ was higher in shade plants (Figures 3K–O). Shade plant requires a larger Φ_f,d_ for energy dissipation due to lower PSII activity and the limited NPQ. Φ_f,d_ reflects the excess excitation energy released by singlet chlorophyll molecule (1Chl) through the triplet pathway (Verhoeven et al., 1997). When light energy is in excess, 1Chl is transited to the triplet chlorophyll molecule (3Chl), and 3Chl interacts with O^2^ to form toxic reactive oxygen species (ROS) (O^2–^, H_2_O_2_, etc.). These ROS might cause damage to the photosynthetic apparatus (Xu et al., 1999). The photochemical efficiency and non-photochemical heat dissipation capacity of shade plants are relatively lower due to the decelerated *F*_v_′/*F*_m_′ and NPQ, and shade plant is more likely to cause photooxidative damage when suddenly exposed to high light. In addition, the rate of PSII recovery depends on the intensity of incident light and the dissipation of light energy (Takahashi and Murata, 2008). When exposed to transient high light, sun plants showed higher Φ_PSII_ than shade plants after the dark recovery due to greater photochemical efficiency and non-photochemical dissipation (Figure 3). Similar results have been observed in *Haberlea rhodopensis* when exposed to transient light (Durgud et al., 2018). Therefore, the light energy captured by sun plants is dissipated mainly through Φ_PSII_ and Φ_NPQ_, while that by shade plants is from Φ_f,d_.

### Greater Cyclic Electron Flow Is Initiated to Confer Photoprotection in Sun Plants Under Transient High Light, While Larger Y(ND) Protect PSI in Shade Plants

Photochemistry of PSII and Y(I) reflect the actual capture efficiency of primary light energy of PSII and PSI and are relative indexes of the speed of photosynthetic electron transport rate (Zhang et al., 2015). They were lower in shade plants than in sun plants when they were suddenly exposed to transient light (Figures 3A–E, 4A–E), suggesting that electron transport rates in PSI and PSII [ETR(I) and ETR(II)] were lower in shade plants (Figures 6A–J). Elevated Y(ND) in shade plants led to reduced electron transport to PSI, resulting in reductions in ETR(I) and ETR(II) (Figures 6A–J). Meanwhile, larger Y(ND) indicates a higher proportion of oxidation state P700, which dissipates excess light energy at the PSI to protect itself (Klughammer and Schreiber, 2008; Stirbet and Govindjee, 2011). Y(NA) is lower on the receptor side of the shade plants, indicating that PSI is inhibited due to the over-reduction on the receptor side of PSI (Nuijs et al., 1986). The higher Y(NA) can lead to a lower rate of linear electron transport (Stirbet and Govindjee, 2011). However, the ETR(I) and ETR(II) of sun plant *E. breviscapus* remained higher when Y(NA) was larger, which was associated with a higher Y(I) and a lower Y(ND), resulting in an increased electron transfer rate and an increase in the number of electrons transferred to PSI. PSI is more stable than PSII, and the rapid electron transport of PSI in sun plants shows that the increase in Y(NA) is not caused by photodamage (Ballottari et al., 2007). The larger PSI activity (Figure 10) and Φ_PSII_ of sun plants led to larger Y(I), and thus this might confront high excitation pressure at the PSI receptor, increasing Y(NA). Therefore, sun plants enhance Y(NA) to avoid photodamage to PSI and to maintain the original photochemical efficiency. Meanwhile, low Y(ND) in sun plants promoted electron transport to PSI, inducing the increases in ETR(I) and ETR(II) (Figures 6A–J). The results obtained in this study showed that sun plants have a higher quantum yield and electron transport rate to dissipate light energy when they are suddenly exposed to transient light, while shade plants protect PSI by enhancing Y(ND).

As NPQ is triggered by a proton gradient across the thylakoid membrane (ΔpH), the capacity of NPQ to protect PSII is limited as ΔpH has a threshold (Horton and Ruban, 1992; Ruban and Belgio, 2014; Giovagnetti et al., 2015). Thus, in addition to NPQ, CEF around PSI is the other major protective mechanism of the photosystem under excess light energy (Ivanov et al., 2017; Pinnola and Bassi, 2018). CEF can compensate for ATP deficiency by transferring electrons from PSI to PQ, thus allowing proton transfer to the cystoid lumen without reducing NADP^+^ and establishing a high trans-cystoid proton gradient (ΔpH) (Munekage et al., 2002; Yamori and Shikanai, 2016). In this study, CEF was rapidly elevated in shade plant *P. notoginseng*, intermediate plant *P. kingianum*, and sun plant *E. breviscapus* when they were suddenly exposed to transient light. However, the CEF of sun plants was larger than shade plants under transient high light conditions (Figures 6K–O). This may be related to the greater slope of post-illumination (Figure 7), P700 dark reduction rate (Figure 8), and PQ pool (Figure 9). The larger slope of post-illumination and higher P700 dark reduction rate promote the rate of cyclic electron transport, and the higher PQ pool accumulates more electron carriers (Lu et al., 2019), resulting in a higher CEF of sun plants to protect the photosystem from damage. A higher *P*_m_ indicates a higher amplitude of P700+ in sun plants (Figure 10), which also causes a larger CEF. The larger qP (Figures 2F–J) in sun plants led to a larger number of primary electron receptors in the reduced state when suddenly exposed to transient light. This can be used as a signal to activate CEF, thus promoting the formation of ΔpH and alleviating the over-reduction of PSI electron carriers (Kramer et al., 2004; Goltsev et al., 2016; Yang et al., 2019b). Furthermore, higher values of T_30_, T_50_, and T_90_ in sun plant *E. breviscapus* (Table 3) indicated that *E. breviscapus* is relatively insensitive to changes in light intensity. In summary, the CEF of sun plants is larger under transient high light conditions, while shade plants more quickly respond to changes in light intensity.

The increase in CEF (Figures 6K–O) was accompanied by a significant increase in NPQ (Figures 2A–E), and there was a significant positive correlation between the two parameters (Figure 5A). In addition, ETR(II) had a significant positive correlation with NPQ (Figure 5C), suggesting that the ΔpH produced by ETR(II) and CEF activates the NPQ pathway, dissipating excessive excitation energy and reducing damage to photosynthetic apparatus (Huang et al., 2011; Li and Zhang, 2015; Fan et al., 2016). The correlation between ETR(II) and NPQ is higher than that between CEF and NPQ, indicating that ETR(II) has a higher contribution to the buildup of NPQ. It has been shown that ΔpH produced by CEF not only increases ATP synthesis but also balances the rate of ATP and NADPH generated by linear electron transport (Shikanai, 2007; Huang et al., 2011). These would promote the oxidation of P700 to prevent over-reduction of the PSI receptor side (Shikanai, 2007; Huang et al., 2011; Li and Zhang, 2015) and inhibit photodamage to PSII by protecting the activity of the oxygen-evolving complex (OEC) (Takahashi et al., 2009). Shade plants have a greater ratio of oxidation state P700 (P700^+^) [Y(ND)] under transient light (Figures 4F–J), and CEF has a correlation with Y(ND) in the shade plants (Figure 5B). This indicates that the close cooperation between CEF and NPQ increases the P700^+^ ratio in shade plants, thus preventing the over-reduction of the photosynthetic electron transport chain (Li and Zhang, 2015). Meanwhile, such a higher ΔpH in shade plants owing to lower activity of chloroplast ATP synthase not only helps the photoprotection for OEC in PSII but also fine-tunes the redox state of PSI. It has been reported that *Eupatorium adenophorum* and *Cersus cerasoides* adapt to a dynamic high-light environment by enhancing CEF (Wang et al., 2004; Yang et al., 2019b). This is consistent with the greater CEF recorded in sun plant *E. breviscapus* due to its growth under full sunlight, while CEF in shade plants is lower due to their growth under shade. In short, the greater CEF in sun plants makes them have vigorous photoprotection under transient high light.

## Conclusion

**FIGURE 13:**
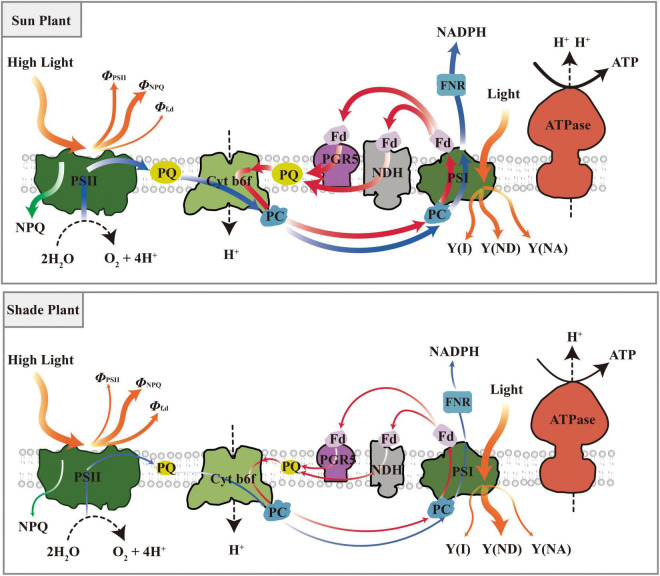
A model was proposed for the photoprotection strategy of shade plant and sun plant under dynamic light conditions. Sun plants had higher NPQ and CEF for photoprotection under transient high light, and mainly dissipate energy through Φ_PSII_ and Φ_NPQ_. While shade plants have a higher Φ_f,d_ and a larger the heat dissipation efficiency of PSI donor. Blue arrows represent linear electron transport, red arrows represent cyclic electron transport, orange arrows represent distribution of light energy, green arrows represent NPQ.

Plants rapidly activate NPQ and CEF to dissipate excess light energy and operate photoprotection when suddenly exposed to dynamic light. NPQ is more active and CEF is initiated more readily in shade plants under transient light. Sun plants show higher NPQ and CEF for photoprotection under transient high light, which shows that they can optimize their performance in high light. In addition, sun plants mainly dissipate energy through Φ_PSII_ and Φ_NPQ_ under transient dynamic light and have a larger PSI quantum yield. While shade plants have a higher Φ_f,d_ and a larger heat dissipation efficiency of PSI donor. A model has been proposed for the photoprotection strategy of shade plants and sun plants under dynamic light conditions (Figure 13). In short, the photochemical efficiency and photoprotective capacity are greater in sun plants under transient dynamic light, while shade plants respond more quickly to transient dynamic light. However, the findings of this study need to be further verified because the investigation was only conducted on the three species, namely, shade-tolerant species *P. notoginseng*, intermediate species *P. kingianum*, and sun-demanding species *E. breviscapus*.

## Data Availability Statement

The original contributions presented in the study are included in the article/Supplementary Material, further inquiries can be directed to the corresponding authors.

## Author Contributions

J-WC, S-PS, J-YZ, ZC, and H-MW conceived and designed the study. S-PS and JH performed the experiments and analyzed the data. S-PS, J-WC, and J-YZ wrote the manuscript. All authors contributed to and edited the final manuscript.

## Conflict of Interest

The authors declare that the research was conducted in the absence of any commercial or financial relationships that could be construed as a potential conflict of interest.

## Publisher’s Note

All claims expressed in this article are solely those of the authors and do not necessarily represent those of their affiliated organizations, or those of the publisher, the editors and the reviewers. Any product that may be evaluated in this article, or claim that may be made by its manufacturer, is not guaranteed or endorsed by the publisher.

## References

[B1] Augspurger C. K. (1984). Light requirements of neotropical tree seedlings: a comparative study of growth and survival. *J. Ecol.* 72 777–795. 10.2307/2259531

[B2] Ballottari M. Dall”Osto L. Morosinotto T. Bassi R. (2007). Contrasting behavior of higher plant photosystem I and II antenna systems during acclimation. *J. Biol. Chem.* 282 8947–8958. 10.1074/jbc.M606417200 17229724

[B3] Boardman N. K. (1977). Comparative photosynthesis of sun and shade plants. *Plant Physiol.* 28 355–377. 10.1146/annurev.pp.28.060177.002035

[B4] Burrows P. A. Sazanov L. A. Svab Z. Maliga P. Nixon P. J. (1998). Identification of a functional respiratory complex in chloroplasts through analysis of tobacco mutants containing disrupted plastid NDH genes. *EMBO J.* 17 868–876. 10.1093/emboj/17.4.868 9463365 PMC1170436

[B5] Chen J. W. Kuang S. B. Long G. Q. Meng Z. G. Li L. G. Chen Z. J. (2014). Steady-state and dynamic photosynthetic performance and nitrogen partitioning in the shade-demanding plant *Panax notoginseng* under different levels of growth irradiance. *Acta Physiol. Plant.* 36 2409–2420. 10.1007/s11738-014-1614-9

[B6] Chen J. W. Kuang S. B. Long G. Q. Yang S. C. Meng Z. G. Li L. G. (2016). Photosynthesis, light energy partitioning, and photoprotection in the shade-demanding species *Panax notoginseng* under high and low level of growth irradiance. *Funct. Plant Biol.* 43 479–491. 10.1071/FP15283 32480478

[B7] DalCorso G. Pesaresi P. Masiero S. Aseeva E. Schünemann D. Finazzi G. (2008). A complex containing PGRL1 and PGR5 is involved in the switch between linear and cyclic electron flow in *Arabidopsis*. *Cell* 132 273–285. 10.1016/j.cell.2007.12.028 18243102

[B8] Demmig-Adams B. Adams W. W. III Barker D. H. Logan B. A. Bowling D. R. Verhoeven A. S. (1996). Using chlorophyll fluorescence to assess the fraction of absorbed light allocated to thermal dissipation of excess excitation. *Physiol. Plant*. 98 253–264. 10.1034/j.1399-3054.1996.980206.x 11841302

[B9] Demmig-Adams B. Cohu C. M. Muller O. Adams W. W. III (2012). Modulation of photosynthetic energy conversion efficiency in nature: from seconds to season. *Photosynth. Res.* 113 75–88. 10.1007/s11120-012-9761-6 22790560

[B10] Durgud M. Gupta S. Ivanov I. Omidbakhshfard A. Benina M. Alseekh S. (2018). Molecular mechanisms preventing senescence in response to prolonged darkness in a desiccation-tolerant plant. *Plant Physiol.* 177 1319–1338. 10.1104/pp.18.00055 29789435 PMC6053018

[B11] Ehleringer J. Pearcy R. W. (1983). Variation in quantum yield for CO_2_ uptake among C3 and C4 plants. *Plant Physiol.* 73 555–559. 10.1104/pp.73.3.555 16663257 PMC1066505

[B12] Fan D. Y. Fitzpatrick D. Oguchi R. Ma W. Kou J. Chow W. S. (2016). Obstacles in the quantification of the cyclic electron flux around photosystem I in leaves of C3 plants. *Photosynth. Res.* 129 239–251. 10.1007/s11120-016-0223-4 26846653

[B13] Farooq S. Chmeliov J. Wientjes E. Koehorst R. Bader A. Valkunas L. (2018). Dynamic feedback of the photosystem II reaction centre on photoprotection in plants. *Nat. Plants* 4 225–231. 10.1038/s41477-018-0127-8 29610535

[B14] Feng Y. L. Cao K. F. Feng Z. L. (2002). Effect of growth light intensity on the photosynthetic apparatus in four tropical rainforest tree species seedlings. *Physiol. Mol. Biol. Plants* 28 153–160. 10.1007/s11769-002-0026-8

[B15] Feng Y. L. Feng Z. L. Cao K. F. (2001). The protection against photodamage in *Amomum villosum Lour*. *Acta Phytophysiol. Sin.* 27 483–488. 10.3321/j.issn:1671-3877.2001.06.008 30704229

[B16] Garcia-Molina A. Leister D. (2020). Accelerated relaxation of photoprotection impairs biomass accumulation in *Arabidopsis*. *Nat. Plants* 6 9–12. 10.1038/s41477-019-0572-z 31907400

[B17] Genty B. Briantais J. M. Baker N. R. (1989). The relationship between the quantum yield of photosynthetic electron transport and quenching of chlorophyll fluorescence. *BBA-Gen. Subj.* 990 87–92. 10.1016/S0304-4165(89)80016-9

[B18] Gerganova M. T. Faik A. K. Velitchkova M. Y. (2019). Acquired tolerance of the photosynthetic apparatus to photoinhibition as a result of growing *Solanum lycopersicum* at moderately higher temperature and light intensity. *Funct. Plant Biol.* 46 555–566. 10.1071/FP18264 30940333

[B19] Giovagnetti V. Ware M. A. Ruban A. V. (2015). Assessment of the impact of photosystem I chlorophyll fluorescence on the pulse-amplitude modulated quenching analysis in leaves of *Arabidopsis thaliana*. *Photosynth. Res.* 125 179–189. 10.1007/s11120-015-0087-z 25613087

[B20] Goltsev V. N. Kalaji H. M. Paunova M. Bąba W. Horaczek T. Mojski J. (2016). Variable chlorophyll fluorescence and its use for assessing physiological condition of plant photosynthetic apparatus. *Russ. J. Plant Physiol.* 63 869–893. 10.1134/S1021443716050058

[B21] Hallik L. Niinemets Ü Kull O. (2011). Photosynthetic acclimation to light in woody and herbaceous species: a comparison of leaf structure, pigment content and chlorophyll fluorescence characteristics measured in the field. *Plant Biol.* 14 88–99. 10.1111/j.1438-8677.2011.00472.x 21972867

[B22] Hendrickson L. Furbank R. T. Chow W. S. (2004). A simple alternative approach to assessing the fate of absorbed light energy using chlorophyll fluorescence. *Photosynth. Res.* 82 73–81. 10.1023/B:PRES.0000040446.87305.f4 16228614

[B23] Horton P. Ruban A. V. (1992). Regulation of Photosystem II. *Photosynth. Res.* 34 375–385. 10.1007/BF00029812 24408833

[B24] Horton P. Johnson M. P. Perez-Bueno M. L. Kiss A. Z. Ruban A. V. (2008). Photosynthetic acclimation: does the dynamic structure and macro-organisation of photosystem II in higher plant grana membranes regulatelight harvesting states? *FEBS J.* 275 1069–1079. 10.1111/j.1742-4658.2008.06263.x 18318834

[B25] Huang W. Zhang S. B. Cao K. F. (2012a). Physiological role of cyclic electron floe in higher plants. *Plant Sci. J.* 30 100–106. 10.3724/SP.J.1142.2012.10100

[B26] Huang W. Yang S. J. Zhang S. B. Zhang J. L. Cao K. F. (2012b). Cyclic electron flow plays an important role in photoprotection for the resurrection plant *Paraboea rufescens* under drought stress. *Planta* 235 819–828. 10.1007/s00425-011-1544-3 22080919

[B27] Huang W. Zhang S. B. Cao K. F. (2011). Cyclic electron flow plays an important role in photoprotection of tropical trees illuminated at temporal chilling temperature. *Plant Cell Physiol.* 52 297–305. 10.1093/pcp/pcq166 21062868

[B28] Huang W. Zhang S. B. Zhang J. L. Hong H. (2015). Photoinhibition of photosystem I under high light in the shade-established tropical tree species *Psychotria rubra*. *Front. Plant Sci.* 6:801. 10.3389/fpls.2015.00801 26483816 PMC4586421

[B29] Hubbart S. Ajigboye O. O. Horton P. Murchie E. H. (2012). The photoprotective protein PsbS exerts control over CO_2_ assimilation rate in fluctuating light in rice. *Plant J.* 71 402–412. 10.1111/j.1365-313X.2012.04995.x 22413771

[B30] Ikeuchi M. Uebayashi N. Sato F. Endo T. (2014). Physiological functions of PsbS-dependent and PsbS-independent NPQ under naturally fluctuating light conditions. *Plant Cell Physiol.* 55 1286–1295. 10.1093/pcp/pcu069 24850835

[B31] Ishida S. Uebayashi N. Tazoe Y. Ikeuchi M. Homma K. Sato F. (2014). Diurnal and developmental changes in energy allocation of absorbed light at PSII in field-grown rice. *Plant Cell Physiol*. 55, 171–182. 10.1093/pcp/pct169 24259682

[B32] Ivanov A. G. Velitchkova M. Y. Allakhverdiev S. I. Huner N. P. A. (2017). Heat stress-induced effects of photosystem I: an overview of structural and functional responses. *Photosynth. Res*. 133, 17–30. 10.1007/s11120-017-0383-x 28391379

[B33] Jahns P. Holzwarth A. R. (2012). The role of the xanthophyll cycle and of lutein in photoprotection of photosystem II. *BBA-Bioenergetics* 1817 182–193. 10.1016/j.bbabio.2011.04.012 21565154

[B34] Johnson M. P. Ruban A. V. (2010). *Arabidopsis* plants lacking PsbS protein possess photoprotective energy dissipation. *Plant J.* 61 283–289. 10.1111/j.1365-313x.2009.04051.x 19843315

[B35] Johnson M. P. Ruban A. V. (2011). Restoration of rapidly reversible photoprotective energy dissipation in the absence of PsbS protein by enhanced ΔpH. *J. Biol. Chem.* 286 19973–19981. 10.1074/jbc.M111.237255 21474447 PMC3103371

[B36] Kitajima K. (1994). Relative importance of photosynthetic traits and allocation patterns as correlates of seedling shade tolerance of 13 tropical trees. *Oecologia* 98 419–428. 10.1007/BF00324232 28313920

[B37] Klughammer C. Schreiber U. (2008). Saturation pulse method for assessment of energy conversion in PSI. *PAM Appl. Notes* 1 11–14. 10.1093/jxb/erp131 19420284 PMC2718210

[B38] Kornyeyev D. Hendrickson L. (2007). Energy partitioning in photosystem II complexes subjected to photoinhibitory treatment. *Funct. Plant Biol.* 34 214–220. 10.1071/FP06327 32689347

[B39] Kramer D. M. Johnson G. Kiirats O. Edwards E. (2004). New fluorescence parameters for the determination of QA redox state and excitation energy fluxes. *Photosynth. Res.* 79 209–218. 10.1023/B:PRES.0000015391.99477.0d 16228395

[B40] Kubo S. Masumura T. Saito Y. Fukayama H. Suzuki Y. Sugimoto T. (2011). Cyclic electron flow around PSI functions in the photoinhibited rice leaves. *Soil Sci. Plant Nutr.* 57 105–113. 10.1080/00380768.2011.553803

[B41] Li J. W. Zhang S. B. (2015). Differences in the responses of photosystems I and II in *Cymbidium sinense* and *C. tracyanum* to long-term chilling stress. *Front. Plant Sci.* 6:1097. 10.3389/fpls.2015.01097 26779201 PMC4700187

[B42] Li R. Liu G. Z. Lu Y. C. Yang J. W. Guan D. J. Yang S. C. (2019). Evaluation and identification of special composition germplasms in *Erigeron breviscapus*. *Chin. J. Trop. Crops* 40 1713–1722. 10.3969/j.issn.1000-2561.2019.09.007

[B43] Li X. P. BjoÈrkman O. Shih C. Grossman A. R. Rosenquist M. Jansson S. (2000). A pigment-binding protein essential for regulation of photosynthetic light harvesting. *Nature* 403 391–395. 10.1038/35000131 10667783

[B44] Li X. P. Gilmore A. M. Ca?arri S. Bassi R. Golan T. Kramer D. (2004). Regulation of photosynthetic light harvesting involves in trathylakoid lumen pH sensing by the PsbS protein. *J. Biol. Chem.* 279 22866–22874. 10.1074/jbc.M402461200 15033974

[B45] Lichtenthaler H. K. Babani F. (2004). Light adaptation and senescence of the photosynthetic apparatus. Changes in pigment composition, chlorophyll fluorescence parameters and photosynthetic activity. *J. Jpn. Soc. Waste Manag. Experts* 7 68–77. 10.1007/978-1-4020-3218-9_28

[B46] Liu J. Last R. L. (2017). A chloroplast thylakoid lumen protein is required for proper photosynthetic acclimation of plants under fluctuating light environments. *Proc. Natl. Acad. Sci. U. S. A.* 114 E8110–E8117. 10.1073/pnas.1712206114 28874535 PMC5617312

[B47] Lu J. Yin Z. Lu T. Yang X. Liu Y. (2019). Cyclic electron flow modulate the linear electron flow and reactive oxygen species in tomato leaves under high temperature. *Plant Sci.* 292:110387. 10.1016/j.plantsci.2019.110387 32005392

[B48] Major K. M. Dunton K. H. (2002). Variations in light-harvesting characteristics of the seagrass, *Thalassia testudinum*: evidence for photoacclimation. *J. Exp. Mar. Biol. Ecol.* 275 173–189. 10.1016/S0022-0981(02)00212-5

[B49] Maxwell K. Johnson G. N. (2000). Chlorophyll fluorescence-a practical guide. *J. Exp. Bot.* 51 659–668. 10.1093/jexbot/51.345.659 10938857

[B50] Mishanin V. I. Trubitsin B. V. Patsaeva S. V. Ptushenko V. V. Solovchenko A. E. Tikhonov A. N. (2017). Acclimation of shade-tolerant and light-resistant *Tradescantia* species to growth light: chlorophyll a fluorescence, electron transport, and xanthophyll content. *Photosynth. Res.* 133 1–16. 10.1007/s11120-017-0339-1 28176042

[B51] Miyake C. (2010). Alternative electron flows (water-water cycle and cyclic electron flow around PSI) in photosynthesis: molecular mechanisms and physiological functions. *Plant Cell Physiol.* 51 1951–1963. 10.1093/pcp/pcq173 21068108

[B52] Miyake C. Miyata M. Shinzaki Y. Tomizawa K. (2005). CO_2_ response of cyclic electron flow around PSI (CEF-PSI) in tobacco leaves-relative electron fluxes through PSI and PSII determine the magnitude of non-photochemical quenching (NPQ) of Chl fluorescence. *Plant Cell Physiol.* 46 629–637. 10.1093/pcp/pci067 15701657

[B53] Munekage Y. Hojo M. Meurer J. Endo T. Tasaka M. Shikanai T. (2002). PGR5 is involved in cyclic electron flow around photosystem I and is essential for photoprotection in *Arabidopsis*. *Cell* 110 361–371. 10.1016/S0092-8674(02)00867-X 12176323

[B54] Murchie E. H. Niyogi K. K. (2011). Manipulation of photoprotection to improve plant photosynthesis. *Plant Physiol.* 155 86–92. 10.1104/pp.110.168831 21084435 PMC3075776

[B55] Niyogi K. K. Truong T. B. (2013). Evolution of flexible non-photochemical quenching mechanisms that regulate light harvesting in oxygenic photosynthesis. *Curr. Opin. Plant Biol.* 16 307–314. 10.1016/j.pbi.2013.03.011 23583332

[B56] Nuijs A. M. Shuvalov V. A. van Gorkom H. J. M. Plijter J. J. Duysens L. N. M. (1986). Picosecond absorbance difference spectroscopy on the primary reactions and the antenna-excited states in photosystem I particles. *BBA-Bioenergetics* 850 310–318. 10.1016/0005-2728(86)90186-6

[B57] Pérez-Bueno M. L. Johnson M. P. Zia A. Ruban A. V. Horton P. (2008). The Lhcb protein and xanthophyll composition of the light harvesting antenna controls the ΔpH-dependency of non-photochemical quenching in *Arabidopsis thaliana*. *FEBS Lett.* 582 1477–1482. 10.1016/j.febslet.2008.03.040 18396161

[B58] Pinnola A. Bassi R. (2018). Molecular mechanisms involved in plant photoprotection. *Biochem. Soc. Trans*. 46, 467–482. 10.1042/BST20170307 29666217

[B59] Poorter H. Niinemets Ü Ntagkas N. Siebenkäs A. Mäenpää M. Matsubara S. (2019). A meta-analysis of plant responses to light intensity for 70 traits ranging from molecules to whole plant performance. *New Phytol.* 223 1073–1105. 10.1111/nph.15754 30802971

[B60] Popova A. V. Dobrev K. Velitchkova M. Ivanov A. G. (2018). Differential temperature effects on dissipation of excess light energy and energy partitioning in lut2 mutant of *Arabidopsis thaliana* under photoinhibitory conditions. *Photosynth. Res.* 139 367–385. 10.1007/s11120-018-0511-2 29725995

[B61] Rascher U. Nedbal L. (2006). Dynamics of photosynthesis in fluctuating light. *Curr. Opin. Plant Biol*. 9 671–678. 10.1016/j.pbi.2006.09.012 17011815

[B62] Ruban A. V. Belgio E. (2014). The relationship between maximum tolerated light intensity and photoprotective energy dissipation in the photosynthetic antenna: chloroplast gains and losses. *Philos. Trans. R. Soc. B Biol. Sci*. 369:20130222. 10.1098/rstb.2013.0222 24591709 PMC3949387

[B63] Sato R. Ohta H. Masuda S. (2014). Prediction of respective contribution of linear electron ?ow and PGR5-dependent cyclic electron ?ow to non-photochemical quenching induction. *Plant Physiol. Biochem.* 81 190–196. 10.1016/j.plaphy.2014.03.017 24725611

[B64] Satoshi I. Nozomu U. Youshi T. Masahiro I. Koki H. Fumihiko S. (2014). Diurnal and developmental changes in energy allocation of absorbed light at PSII in field-grown rice. *Plant Cell Physiol.* 55 171–182. 10.1093/pcp/pct169 24259682

[B65] Savitch L. V. Barker-Astrom J. Ivanov A. G. Hurry V. Öquist G. Gardeström H. P. (2001). Cold acclimation of *Arabidopsis thaliana* results in incomplete recovery of photosynthetic capacity, associated with an increased reduction of the chloroplast stroma. *Planta* 214 295–303. 10.1007/s004250100622 11800395

[B66] Schneider T. Bolger A. Zeier J. Preiskowski S. Benes V. Trenkamp S. (2019). Fluctuating light interacts with time of day and leaf development stage to reprogram gene expression. *Plant Physiol.* 179 1632–1657. 10.1104/pp.18.01443 30718349 PMC6446761

[B67] Shikanai T. (2007). Cyclic electron transport around photosystem I: genetic approaches. *Annu. Rev. Plant Biol.* 58 199–217. 10.1146/annurev.arplant.58.091406.110525 17201689

[B68] Stirbet A. Govindjee. (2011). On the relation between the Kautsky effect (chlorophyll a fluorescence induction) and Photosystem II: basics and applications of the OJIP fluorescence transient. *J. Photochem. Photobiol. B* 104 236–257. 10.1016/j.jphotobiol.2010.12.010 21295993

[B69] Su W. H. Zhang G. F. Li X. H. Gu F. X. Shi B. L. (2006). Effect of light intensity and light quality on growth and total flavonoid accumulation of *Erigeron breviscapus*. *Chin. Tradit. Herb. Drugs* 37 1244–1247. 10.1360/yc-006-1325 17035196

[B70] Sun Y. Geng Q. Du Y. Yang X. Zhai H. (2017). Induction of cyclic electron flow around photosystem I during heat stress in grape leaves. *Plant Sci.* 256 65–71. 10.1016/j.plantsci.2016.12.004 28167040

[B71] Suorsa M. Jarvi S. Grieco M. Nurmi M. Pietrzykowska M. Rantala M. (2012). PROTON GRADIENT REGULATION5 is essential for proper acclimation of *Arabidopsis* photosystem I to naturally and artificially fluctuating light conditions. *Plant Cell* 24 2934–2948. 10.1105/tpc.112.097162 22822205 PMC3426124

[B72] Susan T. S. Björkman O. (1990). Leaf xanthophyll content and composition in san and shade determined by HPLC. *Photosynth. Res.* 23 331–343. 10.1007/BF00034864 24419657

[B73] Takahashi S. Murata N. (2008). How do environmental stresses accelerate photoinhibition? *Trends Plant Sci.* 13 178–182. 10.1016/j.tplants.2008.01.005 18328775

[B74] Takahashi S. Milward S. E. Fan D. Y. Chow W. S. Badger M. R. (2009). How does cyclic electron flow alleviate photoinhibition in *Arabidopsis*? *Plant Physiol*. 149 1560–1567. 10.1104/pp.108.134122 19118124 PMC2649389

[B75] Takehiro S. Daisuke T. Hiroshi F. Amane M. Chikahiro M. (2014). Repetitive short-pulse light mainly inactivates photosystem I in sunflower leaves. *Plant Cell Physiol.* 55 1184–1193. 10.1093/pcp/pcu061 24793753

[B76] Tausz M. Warren C. R. Adams M. A. (2005). Dynamic light use and protection from excess light in upper canopy and coppice leaves of *Nothofagus cunninghamii* in an old growth, cool temperate rainforest in Victoria, Australia. *New Phytol.* 165 143–156. 10.2307/1514594 15720629

[B77] Tikhonov A. N. (2013). pH-Dependent regulation of electron transport and ATP synthesis in chloroplasts. *Photosynth. Res.* 116 511–534. 10.1007/s11120-013-9845-y 23695653

[B78] Ueda M. Kuniyoshi T. Yamamoto H. Sugimoto K. Shikanai T. (2012). Composition and physiological function of the chloroplast NADH dehydrogenase-like complex in *Marchantia polymorpha*. *Plant J.* 72 683–693. 10.1111/j.1365-313X.2012.05115.x 22862786

[B79] Valladares F. Niinemets Ü (2008). Shade tolerance, a key plant feature of complex nature and consequences. *Annu. Rev. Ecol. Evol. Syst.* 39 237–257. 10.1146/annurev.ecolsys.39.110707.173506

[B80] Valladares F. Chico J. Aranda I. Balaguer L. Dizengremel P. Manrique E. (2002). The greater seedling high-light tolerance of *Quercus robur* over *Fagus sylvatica* is linked to a greater physiological plasticity. *Trees* 16 395–403. 10.1007/s00468-002-0184-4

[B81] Valladares F. Wright S. J. Lasso E. Kitajima K. Pearcy R. W. (2000). Plastic phenotypic response to light of 16 congeneric shrubs from a Panamanian rainforest. *Ecology* 81 1925–1936. 10.2307/177282

[B82] van Kooten O. Snel J. F. H. (1990). The use of chlorophyll fluorescence nomenclature in plant stress physiology. *Photosynth. Res.* 25 147–150. 10.1007/BF00033156 24420345

[B83] Verhoeven A. S. Demmig-Adams B. Adams W. W. III (1997). Enhanced employment of the xanthophyll cycle and thermal energy dissipation in spinach exposed to high light and N stress. *Plant Physiol.* 113 817–824. 10.1109/NOMSW.2010.5486584 12223645 PMC158201

[B84] Vialet-Chabrand S. Matthews J. S. Simkin A. J. Raines C. A. Lawson T. (2017). Importance of fluctuations in light on plant photosynthetic acclimation. *Plant Physiol.* 173 2163–2179. 10.1104/pp.16.01767 28184008 PMC5373038

[B85] Wang F. Wu N. Zhang L. Ahammed G. J. Chen X. Xiang X. (2017). Light signaling-dependent regulation of photoinhibition and photoprotection in tomato. *Plant Physiol.* 176 1311–1326. 10.1104/pp.17.01143 29146776 PMC5813521

[B86] Wang J. F. Feng Y. L. Liang H. Z. (2004). Adaptation of *Eupatorium adenophorum* photosynthetic characteristics to light intensity. *Chin. J. Appl. Ecol.* 15 1373–1377. 10.1088/1009-0630/6/5/011 15573991

[B87] Way D. A. Pearcy R. W. (2012). Sunflecks in trees and forests: from photosynthetic physiology to global change biology. *Tree Physiol.* 32 1066–1081. 10.1093/treephys/tps064 22887371

[B88] Wei Z. Duan F. Sun X. Song X. Zhou W. (2020). Leaf photosynthetic and anatomical insights into mechanisms of acclimation in rice in response to long-term fluctuating light. *Plant Cell Environ.* 44 747–761. 10.1111/pce.13954 33215722

[B89] Xu X. Z. Zhang J. Y. Zhang G. H. Long G. Q. Yang S. C. Chen Z. J. (2018). Effects of light intensity on photosynthetic capacity and light energy allocation in *Panax notoginseng*. *Chin. J. Appl. Ecol.* 29 193–204. 10.13287/j.1001-9332.201801.008 29692028

[B90] Xu Z. F. Luo G. H. Wang A. G. Chen Y. Z. Guo J. Y. (1999). Effects of strong light and active oxygen on photosynthesis in soybean. *Chin. B Bot.* 41 862–866.

[B91] Yamori W. Shikanai T. (2016). Physiological functions of cyclic electron transport around photosystem I in sustaining photosynthesis and plant growth. *Annu. Rev. Plant Biol.* 67 81–106. 10.1146/annurev-arplant-043015-112002 26927905

[B92] Yamori W. Makino A. Shikanai T. (2016). A physiological role of cyclic electron transport around photosystem I in sustaining photosynthesis under fluctuating light in rice. *Sci. Rep.* 6:20147. 10.1038/srep20147 26832990 PMC4735858

[B93] Yang Y. J. Ding X. X. Huang W. (2019a). Stimulation of cyclic electron flow around photosystem I upon a sudden transition from low to high light in two angiosperms *Arabidopsis thaliana* and *Bletilla striata*. *Plant Sci.* 287:110166. 10.1016/j.plantsci.2019.110166 31481226

[B94] Yang Y. J. Zhang S. B. Wang J. H. Huang W. (2019b). Photosynthetic regulation under fluctuating light in field-grown *Cerasus cerasoides*: a comparison of young and mature leaves. *BBA-Bioenergetics* 1860:148073. 10.1016/j.bbabio.2019.148073 31473302

[B95] Yao X. Zhang J. Y. Wan Q. Q. Li Y. R. Shen T. (2018). Potential geographical distribution of *Polygonatum kingianum* and its climatic suitability analysis. *J. Trop. Subtrop. Bot.* 26 439–448. 10.11926/jtsb.3874

[B96] Zhang J. Shuang S. Zhang L. Xie S. Chen J. (2021). Photosynthetic and photoprotective responses to steady-state and fluctuating light in the shade-demanding crop *Amorphophallus xiei* grown in intercropping and monoculture systems. *Front. Plant Sci.* 12:663473. 10.3389/fpls.2021.663473 34093621 PMC8175988

[B97] Zhang Q. H. Zhang J. Y. Cun Z. Chen J. W. (2020). Effcet of light and temperature on photosystem activities of *Panax notoginseng*. *Plant Physiol. J.* 56 1064–1072. 10.13592/j.cnki.ppj.2019.0475

[B98] Zhang T. J. Feng L. Tian X. S. Yang C. H. Gao J. D. (2015). Use of chlorophyll fluorescence and P700 absorbance to rapidly detect glyphosate resistance in goosegrass (*Eleusine indica*). *J. Integr. Agric.* 14 714–723. 10.1016/S2095-3119(14)60869-8

[B99] Zivcak M. Brestic M. Balatova Z. Drevenakova P. Olsovska K. Kalaji H. M. (2013). Photosynthetic electron transport and specific photoprotective responses in wheat leaves under drought stress. *Photosynth. Res.* 117 529–546. 10.1007/s11120-013-9885-3 23860828

[B100] Zivcak M. Brestic M. Kunderlikova K. Sytar O. Allakhverdiev S. I. (2015). Repetitive light pulse-induced photoinhibition of photosystem I severely affects CO_2_ assimilation and photoprotection in wheat leaves. *Photosynth. Res.* 126 449–463. 10.1007/s11120-015-0121-1 25829027

[B101] Zuo Y. M. Yang T. M. Yang W. Z. Yang S. B. Li J. C. Xu Z. L. (2018). Research on photosynthetic physiological characteristics of *Polygonatum kingianum* for three different provenances. *Guangdong Agric. Sci.* 45 25–31. 10.16768/j.issn.1004-874X.2018.08.004

