# Supplementary material for: A Comparison of Photoprotective Mechanism in Different Light-Demanding Plants Under Dynamic Light Conditions
Source: Front Plant Sci. 2022 Apr 6;13:819843. doi: 10.3389/fpls.2022.819843 (PMC9019478; doi:10.3389/fpls.2022.819843)
Supplement: Supplementary file 1 [file Data_Sheet_1.docx]

**TABLE 1** Parameters of the PSII maximum efficiency (*F*_v_^'^/*F*_m_^'^) induction properties in *P. notoginseng* and *P. kingianum* and *E. breviscapus* studied.

| Light intensity | Species | T_30_(S) | T_50_(S) | T_90_(S) | IS_300S_ | IS_600S_ | IS_1200S_ |  |
| --- | --- | --- | --- | --- | --- | --- | --- | --- |
| 50 | *P. notoginseng* | 54.591±2.156 c | 34.450±1.533 c | 7.600±0.651 c | 0.562±0.021 b | 0.595±0.021 b | 0.634±0.028 b |  |
|  | *P. kingianum* | 81.325±2.607 b | 52.485±2.184 b | 13.658±0.661 b | 0.669±0.019 a | 0.697±0.007 a | 0.732±0.007 a |  |
|  | *E. breviscapus* | 118.638±2.336 a | 90.564±3.739 a | 28.161±1.520 a | 0.711±0.032 a | 0.739±0.032 a | 0.773±0.017 a |  |
| 100 | *P. notoginseng* | 50.824±1.333 c | 30.370±0.902 c | 6.290±0.414 b | 0.594±0.010 c | 0.624±0.011 c | 0.674±0.015 c |  |
|  | *P. kingianum* | 72.886±2.127 b | 42.755±1.087 b | 7.215±0.590 b | 0.666±0.010 b | 0.712±0.016 b | 0.743±0.010 b |  |
|  | *E. breviscapus* | 110.704±3.216 a | 82.259±2.586 a | 28.298±1.243 a | 0.804±0.007 a | 0.832±0.003 a | 0.821±0.002 a |  |
| 400 | *P. notoginseng* | 69.108±4.069 c | 39.977±2.686 b | 7.278±1.251 b | 0.564±0.007 a | 0.560±0.007 a | 0.556±0.008 b |  |
|  | *P. kingianum* | 90.283±3.078 b | 50.504±1.648 b | 7.210±0.481 b | 0.595±0.015 a | 0.587±0.015 a | 0.583±0.015 ab |  |
|  | *E. breviscapus* | 146.472±3.464 a | 259.012±164.894 a | 30.643±0.981 a | 0.606±0.021 a | 0.587±0.015 a | 0.605±0.018 a |  |
| 800 | *P. notoginseng* | 52.635±1.346 c | 30.583±0.751 c | 5.006±0.232 b | 0.523±0.016 b | 0.517±0.016 b | 0.512±0.016 b |  |
|  | *P. kingianum* | 85.592±3.090 b | 47.819±1.854 b | 6.372±0.343 b | 0.614±0.009 ab | 0.608±0.009 ab | 0.603±0.009 ab |  |
|  | *E. breviscapus* | 134.591±4.179 a | 80.716±3.065 a | 19.614±1.865 a | 0.575±0.024 a | 0.563±0.024 a | 0.556±0.023 a |  |
| 1600 | *P. notoginseng* | 62.923±5.102 c | 35.838±2.981 c | 5.977±0.754 b | 0.524±0.011 b | 0.516±0.010 b | 0.507±0.010 b |  |
|  | *P. kingianum* | 85.300±4.281 b | 47.626±1.973 b | 6.024±0.641 b | 0.481±0.045 b | 0.469±0.045 b | 0.460±0.044 b |  |
|  | *E. breviscapus* | 177.801±5.399 a | 105.835±3.780 a | 24.588±1.457 a | 0.621±0.003 a | 0.600±0.003 a | 0.592±0.003 a |  |

The data in the table are mean ± SE (*n*=5). Different letters indicate significant differences between materials (P<0.05). T_30_(S): the time required to reach 30% of maximum *F*_v_^'^/*F*_m_^'^, T_50_(S): the time required to reach 50% of maximum *F*_v_^'^/*F*_m_^'^, T_90_(S): the time required to reach 90% of maximum *F*_v_^'^/*F*_m_^'^, IS_300S_: the induction state of different light-demanding plants after 300s light, IS_600S_: the induction state of different light-demanding plants after 600s light, IS_1200S_: the induction state of different light-demanding plants after 1200s light.

**TABLE 2** Parameters of the non-photochemical quenching (NPQ) induction properties in *P. notoginseng* and *P. kingianum* and *E. breviscapus* studied.

| Light intensity | Species | T_30_(S) | T_50_(S) | T_90_(S) | IS_300S_ | IS_600S_ | IS_1200S_ |  |
| --- | --- | --- | --- | --- | --- | --- | --- | --- |
| 50 | *P. notoginseng* | 25.244±1.434 b | 48.923±2.478 b | 132.264±7.702 b | 1.421±0.184 b | 1.105±0.141 b | 0.772±0.112 a |  |
|  | *P. kingianum* | 20.232±0.458 c | 41.969±1.534 b | 134.843±12.926 b | 1.180±0.183 b | 1.047±0.102 b | 0.887±0.192 a |  |
|  | *E. breviscapus* | 54.804±2.321 a | 92.195±2.840 a | 177.870±8.322 a | 2.213±0.113 a | 1.869±0.207 a | 1.137±0.125 a |  |
| 100 | *P. notoginseng* | 25.750±2.550 b | 51.177±4.724 b | 150.935±11.494 a | 1.676±0.070 a | 1.364±0.114 a | 0.900±0.109 a |  |
|  | *P. kingianum* | 22.663±4.051 b | 45.993±7.906 b | 162.940±19.063 a | 1.448±0.107 ab | 0.725±0.096 b | 0.448±0.013 b |  |
|  | *E. breviscapus* | 51.221±2.602 a | 85.553±4.359 a | 154.668±7.941 a | 1.180±0.090 b | 0.503±0.037 b | 0.361±0.016 b |  |
| 400 | *P. notoginseng* | 16.251±1.431 c | 37.491±3.317 c | 292.544±28.944 a | 2.064±0.115 c | 2.114±0.124 c | 2.173±0.135 c |  |
|  | *P. kingianum* | 21.754±0.891 b | 49.721±2.017 b | 349.009±15.753 a | 2.342±0.057 b | 2.473±0.065 b | 2.560±0.081 b |  |
|  | *E. breviscapus* | 31.617±1.632 a | 72.608±3.139 a | 299.924±5.779 a | 2.726±0.043 a | 2.955±0.080 a | 3.074±0.078 a |  |
| 800 | *P. notoginseng* | 13.750±0.837 c | 31.969±1.941 c | 275.164±18.055 c | 2.007±0.055 b | 2.072±0.058 b | 2.140±0.055 b |  |
|  | *P. kingianum* | 19.951±0.889 b | 46.144±2.062 b | 371.379±21.118 b | 2.228±0.177 b | 2.306±0.178 b | 2.379±0.177 b |  |
|  | *E. breviscapus* | 34.806±1.034 a | 79.003±2.722 a | 479.641±12.339 a | 3.237±0.121 a | 3.468±0.123 a | 3.564±0.145 a |  |
| 1600 | *P. notoginseng* | 17.796±2.184 c | 41.502±5.085 c | 372.313±45.298 b | 2.190±0.090 b | 2.261±0.102 c | 2.362±0.113 c |  |
|  | *P. kingianum* | 25.428±1.251 b | 60.650±4.777 b | 501.392±27.202 a | 2.486±0.084 ab | 2.683±0.090 b | 2.851±0.073 b |  |
|  | *E. breviscapus* | 49.484±1.267 a | 109.465±2.890 a | 590.046±10.902 a | 2.794±0.125 a | 3.228±0.177 a | 3.350±0.168 a |  |

The data in the table are mean ± SE (*n*=5). Different letters indicate significant differences between materials (P<0.05). T_30_(S): the time required to reach 30% of maximum NPQ, T_50_(S): the time required to reach 50% of maximum NPQ, T_90_(S): the time required to reach 90% of maximum NPQ, IS_300S_: the induction state of different light-demanding plants after 300s light, IS_600S_: the induction state of different light-demanding plants after 600s light, IS_1200S_: the induction state of different light-demanding plants after 1200s light.

**TABLE 3** Parameters of the cyclic electron flow (CEF) induction properties in *P. notoginseng* and *P. kingianum* and *E. breviscapus* studied.

| Light intensity | Species | T_30_(S) | T_50_(S) | T_90_(S) | IS_300S_ | IS_600S_ | IS_1200S_ |  |
| --- | --- | --- | --- | --- | --- | --- | --- | --- |
| 50 | *P. notoginseng* | 7.676±0.546 b | 17.150±0.812 b | 67.674±0.416 b | 4.020±0.196 a | 3.380±0.128 a | 2.520±0.102 a |  |
|  | *P. kingianum* | 5.135±0.367 b | 10.968±0.478 c | 71.925±1.071 b | 3.480±0.146 b | 2.820±0.073 b | 2.120±0.153 b |  |
|  | *E. breviscapus* | 46.453±1.288 a | 77.422±2.146 a | 135.559±3.947 a | 2.320±0.124 c | 1.580±0.128 c | 1.040±0.093 c |  |
| 100 | *P. notoginseng* | 6.733±0.548 c | 13.728±0.939 c | 53.766±1.722 c | 7.040±0.140 a | 6.920±0.150 a | 6.360±0.172 a |  |
|  | *P. kingianum* | 11.561±0.730 b | 22.571±1.222 b | 63.560±1.697 b | 7.780±0.262 a | 6.740±0.262 a | 4.880±0.242 b |  |
|  | *E. breviscapus* | 59.390±1.718 a | 98.783±1.901 a | 176.769±4.232 a | 7.060±0.343 a | 5.600±0.167 b | 4.480±0.136 b |  |
| 400 | *P. notoginseng* | 5.192±0.172 b | 10.486±0.413 c | 29.296±1.211 c | 5.920±0.107 c | 6.840±0.204 c | 10.420±0.599 b |  |
|  | *P. kingianum* | 6.357±0.443 b | 13.126±0.824 b | 52.286±2.008 b | 11.700±1.233 b | 9.800±0.445 b | 11.400±0.661 b |  |
|  | *E. breviscapus* | 19.705±0.641 a | 36.905±0.500 a | 105.052±3.298 a | 17.940±0.783 a | 27.340±0.787 a | 31.020±1.360 a |  |
| 800 | *P. notoginseng* | 5.129±0.255 c | 7.377±0.422 c | 33.122±2.784 c | 6.060±0.586 c | 8.960±0.571 b | 11.580±0.731 c |  |
|  | *P. kingianum* | 9.196±0.331 a | 19.344±0.718 a | 78.431±1.831 a | 17.420±1.391 b | 18.560±0.539 a | 22.620±1.273 b |  |
|  | *E. breviscapus* | 7.826±0.307 b | 14.955±0.398 b | 58.349±2.764 b | 23.740±1.429 a | 20.180±0.862 a | 30.300±1.455 a |  |
| 1600 | *P. notoginseng* | 1.922±0.116 b | 5.192±0.126 c | 20.327±1.352 b | 17.980±1.565 b | 14.120±1.012 c | 16.140±1.148 b |  |
|  | *P. kingianum* | 2.676±0.184 b | 6.146±0.269 b | 35.087±1.797 a | 20.220±1.399 b | 20.320±1.693 b | 20.540±1.869 b |  |
|  | *E. breviscapus* | 5.741±0.487 a | 8.331±0.290 a | 24.894±1.795 b | 31.000±1.404 a | 35.820±2.038 a | 51.580±1.306 a |  |

The data in the table are mean ± SE (*n*=5). Different letters indicate significant differences between materials (P<0.05). T_30_(S): the time required to reach 30% of maximum CEF, T_50_(S): the time required to reach 50% of maximum CEF, T_90_(S): the time required to reach 90% of maximum CEF, IS_300S_: the induction state of different light-demanding plants after 300s light, IS_600S_: the induction state of different light-demanding plants after 600s light, IS_1200S_: the induction state of different light-demanding plants after 1200s light.
