# Supplementary material for: A Comparison of Photoprotective Mechanism in Different Light-Demanding Plants Under Dynamic Light Conditions
Source: Front Plant Sci. 2022 Apr 6;13:819843. doi: 10.3389/fpls.2022.819843 (PMC9019478; doi:10.3389/fpls.2022.819843)

**SUPPORTING INFORMATION**

Figure S1 The schematic diagram of calculate parameters of *F*_v_^'^/*F*_m_^'^ induction-related traits.


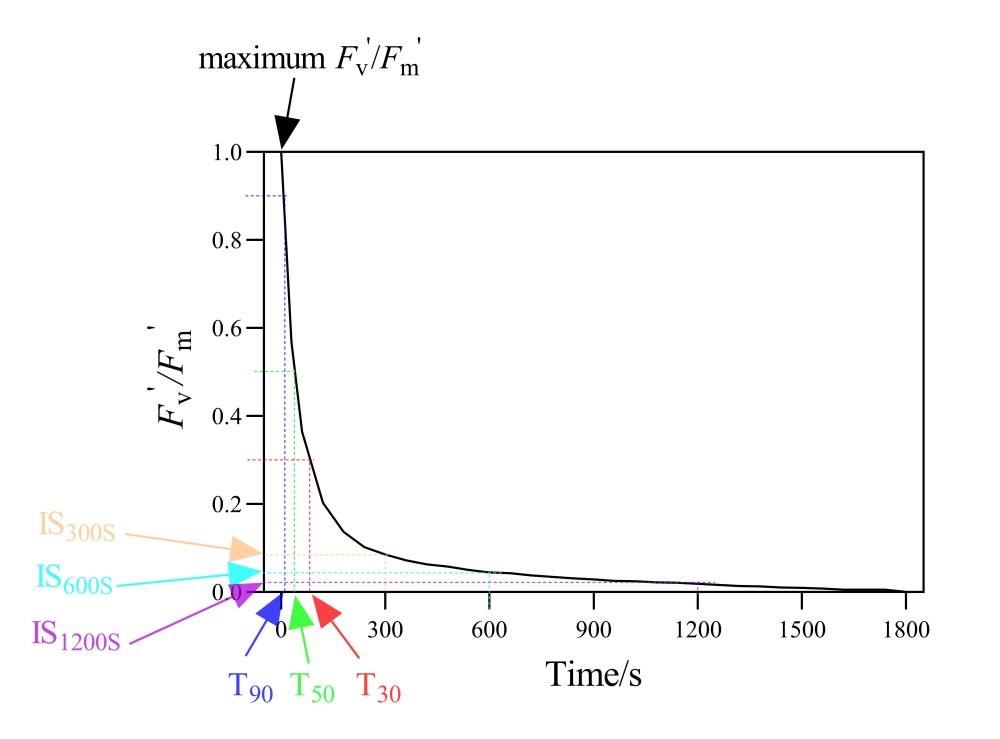

Supplement: Supplementary file 2 [file Data_Sheet_2.docx]
